# High-threshold and low-overhead fault-tolerant quantum memory

**DOI:** 10.1038/s41586-024-07107-7

**Published:** 2024-03-27

**Authors:** Sergey Bravyi, Andrew W. Cross, Jay M. Gambetta, Dmitri Maslov, Patrick Rall, Theodore J. Yoder

**Affiliations:** 1 IBM Quantum, IBM T.J. Watson Research Center, Yorktown Heights, NY USA; 2 IBM Quantum, MIT-IBM Watson AI Laboratory, Cambridge, MA USA

**Keywords:** Quantum information, Computer science, Theoretical physics

## Abstract

The accumulation of physical errors^1–3^ prevents the execution of large-scale algorithms in current quantum computers. Quantum error correction^4^ promises a solution by encoding *k* logical qubits onto a larger number *n* of physical qubits, such that the physical errors are suppressed enough to allow running a desired computation with tolerable fidelity. Quantum error correction becomes practically realizable once the physical error rate is below a threshold value that depends on the choice of quantum code, syndrome measurement circuit and decoding algorithm^5^. We present an end-to-end quantum error correction protocol that implements fault-tolerant memory on the basis of a family of low-density parity-check codes^6^. Our approach achieves an error threshold of 0.7% for the standard circuit-based noise model, on par with the surface code^7–10^ that for 20 years was the leading code in terms of error threshold. The syndrome measurement cycle for a length-*n* code in our family requires *n* ancillary qubits and a depth-8 circuit with CNOT gates, qubit initializations and measurements. The required qubit connectivity is a degree-6 graph composed of two edge-disjoint planar subgraphs. In particular, we show that 12 logical qubits can be preserved for nearly 1 million syndrome cycles using 288 physical qubits in total, assuming the physical error rate of 0.1%, whereas the surface code would require nearly 3,000 physical qubits to achieve said performance. Our findings bring demonstrations of a low-overhead fault-tolerant quantum memory within the reach of near-term quantum processors.

## Abstract (web summary)

An end-to-end quantum error correction protocol that implements fault-tolerant memory on the basis of a family of low-density parity-check codes shows the possibility of low-overhead fault-tolerant quantum memory within the reach of near-term quantum processors.

## Main

Quantum computing attracted attention due to its ability to offer asymptotically faster solutions to a set of computational problems compared to the best known classical algorithms^5^. It is believed that a functioning scalable quantum computer may help solve computational problems in such areas as scientific discovery, materials research, chemistry and drug design, to name a few^11–14^.

The main obstacle to building a quantum computer is the fragility of quantum information, owing to various sources of noise affecting it. As isolating a quantum computer from external effects and controlling it to induce a desired computation are in conflict with each other, noise appears to be inevitable. The sources of noise include imperfections in qubits, materials used, controlling apparatus, state preparation and measurement errors and a variety of external factors ranging from local man-made, such as stray electromagnetic fields, to those inherent to the Universe, such as cosmic rays. See ref. ^15^ for a summary. Whereas some sources of noise can be eliminated with better control^16^, materials^17^ and shielding^18–20^, several other sources appear to be difficult if at all possible to remove. The last kind can include spontaneous and stimulated emission in trapped ions^1,2^, and the interaction with the bath (Purcell effect)^3^ in superconducting circuits—covering both leading quantum technologies. Thus, error correction becomes a key requirement for building a functioning scalable quantum computer.

The possibility of quantum fault tolerance is well-established^4^. Encoding a logical qubit redundantly into many physical qubits enables one to diagnose and correct errors by repeatedly measuring syndromes of parity-check operators. However, error correction is only beneficial if the hardware error rate is below a certain threshold value that depends on a particular error correction protocol. The first proposals for quantum error correction, such as concatenated codes^21–23^, focused on demonstrating the theoretical possibility of error suppression. As understanding of quantum error correction and the capabilities of quantum technologies matured, the focus shifted to finding practical quantum error correction protocols. This resulted in the development of the surface code^7–10^ that offers a high error threshold close to 1%, fast decoding algorithms and compatibility with the existing quantum processors relying on two-dimensional (2D) square lattice qubit connectivity. Small examples of the surface code with a single logical qubit have already been demonstrated experimentally by several groups^24–28^. However, scaling up the surface code to 100 or more logical qubits would be prohibitively expensive due to its poor encoding efficiency. This spurred interest in more general quantum codes known as low-density parity-check (LDPC) codes^6^. Recent progress in the study of LDPC codes suggests that they can achieve quantum fault tolerance with a much higher encoding efficiency^29^. Here, we focus on the study of LDPC codes, as our goal is to find quantum error correction codes and protocols that are both efficient and possible to demonstrate in practice, given the limitations of quantum computing technologies.

A quantum error correcting code is of LDPC type if each check operator of the code acts only on a few qubits and each qubit participates in only a few checks. Several variants of the LDPC codes have been proposed recently including hyperbolic surface codes^30–32^, hypergraph product^33^, balanced product codes^34^, two-block codes based on finite groups^35–38^ and quantum Tanner codes^39,40^. The latter were shown^39,40^ to be asymptotically ‘good’ in the sense of offering a constant encoding rate and linear distance: a parameter quantifying the number of correctable errors. By contrast, the surface code has an asymptotically zero encoding rate and only square-root distance. Replacing the surface code with a high-rate, high-distance LDPC code could have major practical implications. First, the fault-tolerance overhead (the ratio between the number of physical and logical qubits) could be reduced notably. Second, high-distance codes show a very sharp decrease in the logical error rate: as the physical error probability crosses the threshold value, the amount of error suppression achieved by the code can increase by orders of magnitude even with a small reduction of the physical error rate. This feature makes high-distance LDPC codes attractive for near-term demonstrations that are likely to operate in the near-threshold regime. However, it was previously believed that outperforming the surface code for realistic noise models including memory, gate and state preparation and measurement errors may require very large LDPC codes with more than 10,000 physical qubits^31^.

Here we present several concrete examples of high-rate LDPC codes with a few hundred physical qubits equipped with a low-depth syndrome measurement circuit, an efficient decoding algorithm and a fault-tolerant protocol for addressing individual logical qubits. These codes show an error threshold close to 0.7%, show excellent performance in the near-threshold regime and offer a 10 times reduction of the encoding overhead compared with the surface code. Hardware requirements for realizing our error correction protocols are relatively mild, as each physical qubit is coupled by two-qubit gates with only six other qubits. Although the qubit connectivity graph is not locally embeddable into a 2D grid, it can be decomposed into two planar degree-3 subgraphs. As we argue below, such qubit connectivity is well suited for architectures based on superconducting qubits.

**Fig. 1:**
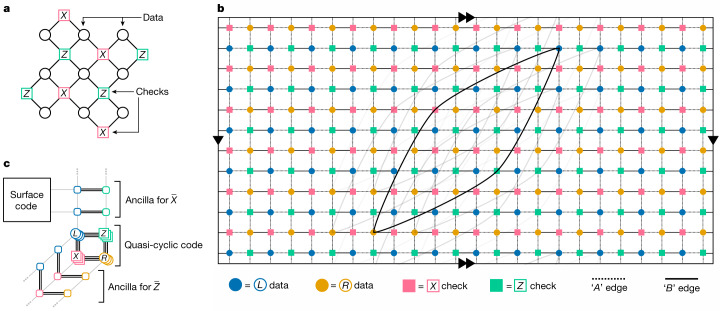
Tanner graphs of surface and BB codes. **a**, Tanner graph of a surface code, for comparison. **b**, Tanner graph of a BB code with parameters [[144, 12, 12]] embedded into a torus. Any edge of the Tanner graph connects a data and a check vertex. Data qubits associated with the registers *q*(*L*) and *q*(*R*) are shown by blue and orange circles. Each vertex has six incident edges including four short-range edges (pointing north, south, east and west) and two long-range edges. We only show a few long-range edges to avoid clutter. Dashed and solid edges indicate two planar subgraphs spanning the Tanner graph, see the Methods. **c**, Sketch of a Tanner graph extension for measuring $\bar{Z}$ and $\bar{X}$ following ref. ^50^, attaching to a surface code. The ancilla corresponding to the $\bar{X}$ measurement can be connected to a surface code, enabling load-store operations for all logical qubits by means of quantum teleportation and some logical unitaries. This extended Tanner graph also has an implementation in a thickness-2 architecture through the *A* and *B* edges (Methods).

Our codes are a generalization of bicycle codes proposed by MacKay et al.^41^ and studied in more depth in refs. ^35,36,42^. We named our codes bivariate bicycle (BB) because they are based on bivariate polynomials, as detailed in the Methods. These are stabilizer codes of the Calderbank–Shor–Steane (CSS) type^43,44^ that can be described by a collection of six-qubit check (stabilizer) operators composed of Pauli *X* and *Z*. At a high level, a BB code is similar to the two-dimensional toric code^7^. In particular, physical qubits of a BB code can be laid out on a two-dimensional grid with periodic boundary conditions such that all check operators are obtained from a single pair of *X* and *Z* checks by applying horizontal and vertical shifts of the grid. However, in contrast to the plaquette and vertex stabilizers describing the toric code, check operators of BB codes are not geometrically local. Furthermore, each check acts on six qubits rather than four qubits. We will describe the code by a Tanner graph *G* such that each vertex of *G* represents either a data qubit or a check operator. A check vertex *i* and a data vertex *j* are connected by an edge if the *i*th check operator acts non-trivially on the *j*th data qubit (by applying Pauli *X* or *Z*). See Fig. 1a,b for example Tanner graphs of surface and BB codes, respectively. The Tanner graph of any BB code has vertex degree six and graph thickness^29^ equal to two, which means it can be decomposed into two edge-disjoint planar subgraphs (Methods). Thickness-2 qubit connectivity is well suited for superconducting qubits coupled by microwave resonators. For example, two planar layers of couplers and their control lines can be attached to the top and the bottom side of the chip hosting qubits, and the two sides mated.

A BB code with parameters [[*n*, *k*, *d*]] encodes *k* logical qubits into *n* data qubits offering a code distance *d*, meaning that any logical error spans at least *d* data qubits. We divide *n* data qubits into registers *q*(*L*) and *q*(*R*) of size *n*/2 each. Any check acts on three qubits from *q*(*L*) and three qubits from *q*(*R*). The code relies on *n* ancillary check qubits to measure the error syndrome. We divide *n* check qubits into registers *q*(*X*) and *q*(*Z*) of size *n*/2 that collect syndromes of *X* and *Z* types, respectively. In total, the encoding relies on 2*n* physical qubits. The net encoding rate is therefore *r* = *k*/(2*n*). For example, the standard surface code architecture encodes *k* = 1 logical qubit into *n* = *d*^2^ data qubits for a distance-*d* code and uses *n* − 1 check qubits for syndrome measurements. The net encoding rate is *r* ≈ 1/(2*d*^2^), which quickly becomes impractical as one is forced to choose a large code distance, due to, for instance, the physical errors being close to the threshold value. By contrast, BB codes have encoding rate *r* ≫ 1/*d*^2^, see Table 1 for code examples. To the best of our knowledge, all codes shown in Table 1 are new. The distance-12 code [[144, 12, 12]] may be the most promising for near-term demonstrations, as it combines large distance and high net encoding rate *r* = 1/24. For comparison, the distance-11 surface code has a net encoding rate *r* = 1/241. Below, we show that the distance-12 BB code outperforms the distance-11 surface code for the experimentally relevant range of error rates.

**Table 1:** Performance of BB codes

[[*n*, *k*, *d*]]	Net encoding rate, *r*	Circuit-level distance, *d*_circ_	Pseudo-threshold, *p*_0_	*p*_L _(10^−3^)	*p*_L _(10^−4^)
[[72, 12, 6]]	1/12	≤6	0.0048	7 × 10^−5 ^	7 × 10^−8^
[[90, 8, 10]]	1/23	≤8	0.0053	5 × 10^−6^	4 × 10^−10^
[[108, 8, 10]]	1/27	≤8	0.0058	3 × 10^−6^	1 × 10^−10^
[[144, 12, 12]]	1/24	≤10	0.0065	2 × 10^−7^	8 × 10^−13^
[[288, 12, 18]]	1/48	≤18	0.0069	2 × 10^−12^	1 × 10^−22^

Small examples of BB LDPC codes and their performance for the circuit-based noise model. All codes have weight-6 checks, depth-7 syndrome measurement circuit, and the Tanner graph composed of two planar subgraphs. A code with parameters [[*n*, *k*, *d*]] requires 2*n* physical qubits in total and achieves the net encoding rate *r* = *k*/2*n* (we round *r* down to the nearest inverse integer). Circuit-level distance *d*_circ_ is the minimum number of faulty operations in the syndrome measurement circuit required to generate a logical error without triggering any syndromes.

**Fig. 2:**
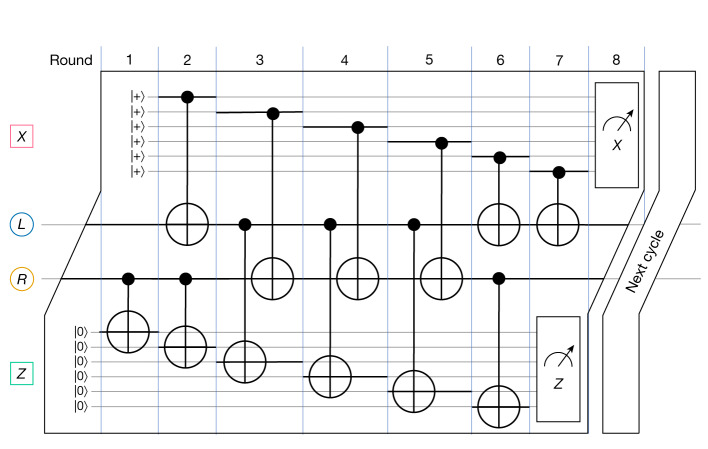
Syndrome measurement circuit. Full cycle of syndrome measurements relying on seven layers of CNOTs. We provide a local view of the circuit that only includes one data qubit from each register *q*(*L*) and *q*(*R*). The circuit is symmetric under horizontal and vertical shifts of the Tanner graph. Each data qubit is coupled by CNOTs with three *X-*check and three *Z-*check qubits: see the Methods for more details.

To prevent the accumulation of errors one must be able to measure the error syndrome frequently enough. This is accomplished by a syndrome measurement circuit that couples data qubits in the support of each check operator with the respective ancillary qubit by a sequence of CNOT gates. Check qubits are then measured revealing the value of the error syndrome. The time it takes to implement the syndrome measurement circuit is proportional to its depth: the number of gate layers composed of non-overlapping CNOTs. As new errors continue to occur while the syndrome measurement circuit is executed, its depth should be minimized. The full cycle of syndrome measurement for a BB code is illustrated on Fig. 2. The syndrome cycle requires only seven layers of CNOTs regardless of the code length. The check qubits are initialized and measured at the beginning and at the end of the syndrome cycle respectively (see the Methods for details). The circuit respects the cyclic shift symmetry of the underlying code.

The full error correction protocol performs *N*_c_ ≫ 1 syndrome measurement cycles and then calls a decoder: a classical algorithm that takes as input the measured syndromes and outputs a guess of the final error on the data qubits. Error correction succeeds if the guessed and the actual error coincide modulo a product of check operators. In this case, the two errors have the same action on any encoded (logical) state. Thus, applying the inverse of the guessed error returns data qubits to the initial logical sate. Otherwise, if the guessed and the actual error differ by a non-trivial logical operator, error correction fails resulting in a logical error. Our numerical experiments are based on the belief propagation with an ordered statistics decoder (BP-OSD) proposed by Panteleev and Kalachev^36^. The original work^36^ described BP-OSD in the context of a toy noise model with memory errors only. Here we show how to extend BP-OSD to the circuit-based noise model, see the Supplementary Information for details. Our approach closely follows refs. ^45–48^.

**Fig. 3:**
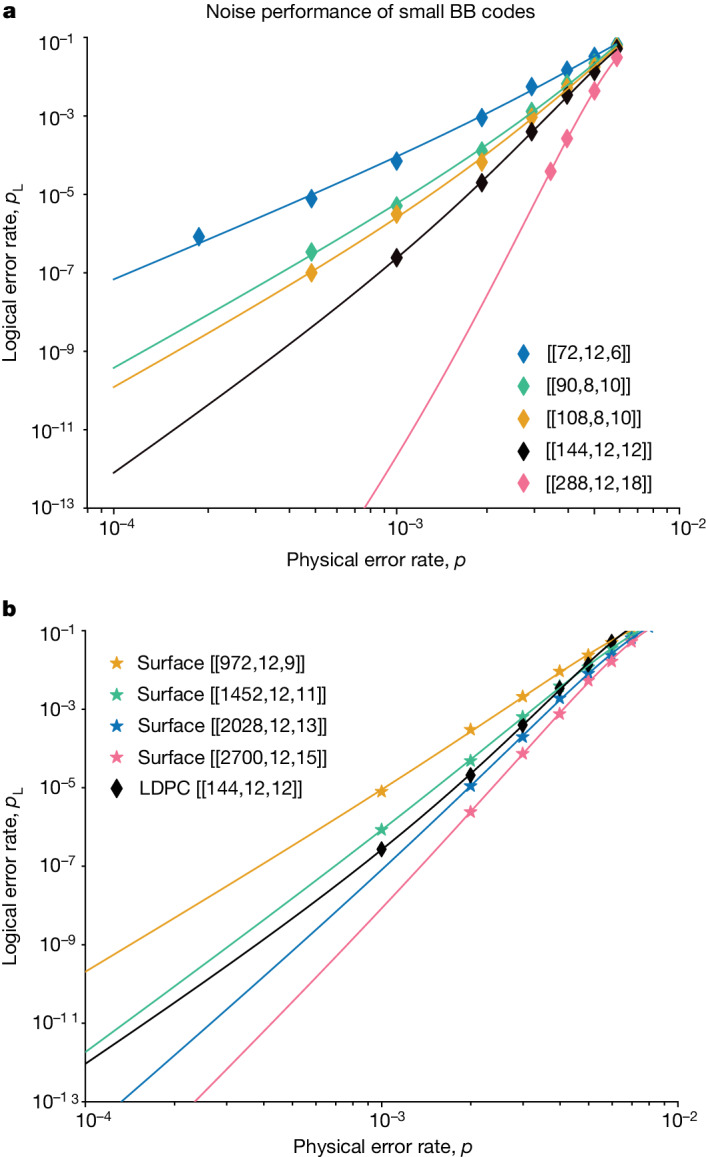
Noise properties of BB codes. **a**, Logical versus physical error rate for small examples of BB LDPC codes. A numerical estimate of *p*_L_ (diamonds) was obtained by simulating *d* syndrome cycles for a distance-*d* code. Most of the data points have error bars roughly equal to *p*_L_/10 due to sampling errors. **b**, Comparison between the BB LDPC code [[144, 12, 12]] and surface codes with 12 logical qubits and distance *d* ∈ {9, 11, 13, 15}. The distance-*d* surface code with 12 logical qubits has the length *n* = 12*d*^2^ because each logical qubit is encoded into a separate *d* × *d* patch of the surface code lattice.

A noisy version of the syndrome measurement circuit may include several types of faulty operations such as memory errors on idle data or check qubits, faulty CNOT gates, qubit initializations and measurements. We consider the circuit-based noise model^10^ in which each operation fails independently with probability *p*. The probability of a logical error *p*_L_ depends on the error rate *p*, details of the syndrome measurement circuits, and the decoding algorithm. Let *P*_L_(*N*_c_) be the logical error probability after performing *N*_c_ syndrome cycles. Define the logical error rate as ${p}_{{\rm{L}}}=1-{(1-{P}_{{\rm{L}}}({N}_{{\rm{c}}}))}^{1/{N}_{{\rm{c}}}}\approx {P}_{{\rm{L}}}({N}_{{\rm{c}}})/{N}_{{\rm{c}}}$. Informally, *p*_L_ can be viewed as the logical error probability per syndrome cycle. Following common practice, we choose *N*_c_ = *d* for a distance-*d* code. Figure 3 shows the logical error rate achieved by codes from Table 1. The logical error rate was computed numerically for *p* ≥ 10^−3^ and extrapolated to lower error rates using a fitting formula (Methods). The pseudo-threshold *p*_0_ is defined as a solution of the break-even equation *p*_L_(*p*)  =  *k*_*p*_. Here *k*_*p*_ is an estimate of the probability that at least one of *k* unencoded qubits suffers from an error. BB codes offer a pseudo-threshold close to 0.7%, see Table 1, which is nearly the same as the error threshold of the surface code^49^ and exceeds the threshold of all high-rate LDPC codes known to the authors.

For example, suppose that the physical error rate is *p* = 10^−3^, which is a realistic goal for near-term demonstrations. Encoding 12 logical qubits using the distance-12 code from Table 1 would offer the logical error rate 2 ×10^−7^, which is enough to preserve 12 logical qubits for nearly 1 million syndrome cycles. The total number of physical qubits required for this encoding is 288. The distance-18 code from Table 1 would require 576 physical qubits whereas suppressing the error rate from 10^−3^ to 2 ×10^−12^ enabling nearly hundred billion syndrome cycles. For comparison, encoding 12 logical qubits into separate patches of the surface code would require more than 3,000 physical qubits to suppress the error rate from 10^−3^ to 10^−7^ (Fig. 3). In this example, the distance-12 BB code offers 10 times saving in the number of physical qubits compared with the surface code.

A proposal for quantum error correction is only useful if the logical qubits are accessible. Fortunately, BB LDPC codes possess the required features to act as a logical memory. As shown in Fig. 1c, extensions of the Tanner graph leveraging techniques by Cohen et al.^50^ enable fault-tolerant measurement operations involving an ancillary surface code. These measurements enable fault-tolerant load-store operations. See the Supplementary Information for details.

Our work highlights key hardware challenges to enable the new codes with superconducting qubits: (1) the development of low-loss second layer in the thickness-2 architecture; (2) the development of qubits that can be coupled to seven connections (six buses and one control line); and (3) the development of long-range couplers.

These are all difficult to solve but not impossible. For the first challenge, we can imagine a small change to the packaging^51^ that was developed for the IBM Quantum Eagle processor^52^. The simplest would be to place the extra buses on the opposite side of the qubit chip. This would require the development of high *Q* through substrate vias that would be part of the coupling buses and as such would require intensive microwave simulation to make sure these through substrate vias could support microwave propagation while not introducing large unwanted crosstalk.

The second challenge is an extension of the number of couplers from the heavy hex lattice arrangement^53^, which is four (three couplers and one control) to seven. The implication of this is that the cross-resonance gate, which has been the core gate used in large quantum systems for the past few years, would not be the path forward. Qubits in cross-resonance gates are not tuneable and as such for a large device with many connections the probability of a energy collisions (not just the qubit levels but also higher levels of the transmon) trends to one very quickly^54^. However, with the tuneable coupler^55,56^ in IBM Quantum Egret and now being developed for the IBM Quantum Heron, this problem no longer exists as qubit frequencies can be designed to be farther apart. This new gate is also similar to the gates used by Google Quantum AI^57^, which have shown that a square lattice arrangement is possible. Extending the coupling map to seven connections will require notable microwave modelling; however, typical transmons have about 60 fF of capacitance and each gate is around 5 fF to get the appropriate coupling strengths to the buses, so it is fundamentally possible to develop this coupling map without altering the long coherence times and stability of transmon qubits.

The final challenge is the most difficult. For the buses that are short enough so that the fundamental mode can be used, the standard circuit quantum electrodynamics model holds. However, to demonstrate the 144-qubit code some of the buses will be long enough that we will require frequency engineering. One way to achieve this is with filtering resonators, and a proof of principle experiment was demonstrated in ref. ^58^.

In summary, we offer a new perspective on how a fault-tolerant quantum memory could be realized using near-term quantum processors with a small qubit overhead. Although these LDPC codes are not geometrically local, qubit connectivity required for syndrome measurements is described by a thickness-2 graph that can be implemented using two planar degree-3 layers of qubit couplers. This is a valid architectural solution for platforms based on superconducting qubits. Numerical simulations performed for the circuit-based noise model indicate that the proposed LDPC codes compare favourably with the surface code in the practically relevant range of error rates *p* ≥ 0.1% offering the same level of error suppression with 10 times reduction in the qubit overhead. Meanwhile, it remains unclear whether our code examples can be scaled up while retaining the high encoding rate in the limit of large code length.

## Methods

### Code construction

We begin with a formal definition of BB codes. Let *I*_*ℓ*_ and *S*_*ℓ*_ be the identity matrix and the cyclic shift matrix of size *ℓ* × *ℓ* respectively. The *i*th row of *S*_*ℓ*_ has a single non-zero entry equal to one at the column $i\,+\,1\,({\rm{mod}}\,\,{\ell })$. For example,

$${S}_{2}=\left[\begin{array}{cc}0 & 1\\ 1 & 0\end{array}\right]\quad {\rm{and}}\quad {S}_{3}=\left[\begin{array}{ccc}0 & 1 & 0\\ 0 & 0 & 1\\ 1 & 0 & 0\end{array}\right].$$

Consider matrices

$$x={S}_{{\ell }}\otimes {I}_{m}\quad {\rm{and}}\quad y={I}_{{\ell }}\otimes {S}_{m}.$$

Note that *xy* = *yx*, *x*^*T*^*x* = *y*^*T*^*y* = *I*_*ℓm*_, and *x*^*ℓ*^ = *y*^*m*^ = *I*_*ℓm*_. A BB code is defined by a pair of matrices

1 $$A={A}_{1}+{A}_{2}+{A}_{3}\quad {\rm{and}}\quad B={B}_{1}+{B}_{2}+{B}_{3}$$

where each matrix *A*_*i*_ and *B*_*j*_ is a power of *x* or *y*. Here and below the addition and multiplication of binary matrices is performed modulo two, unless stated otherwise. Thus, we also assume the *A*_*i*_ are distinct and the *B*_*j*_ are distinct to avoid cancellation of terms. For example, one could choose *A* = *x*^3^ + *y* + *y*^2^ and *B* = *y*^3^ + *x* + *x*^2^. Note that *A* and *B* have exactly three non-zero entries in each row and each column. Furthermore, *AB* = *BA* because *xy* = *yx*. The above data defines a BB quantum code denoted QC(*A*, *B*) with length *n* = 2*ℓm* and check matrices

2 $${H}^{X}=\left[A| B\right]\quad {\rm{and}}\quad {H}^{Z}=\left[{B}^{T}| {A}^{T}\right].$$

Here the vertical bar indicates stacking matrices horizontally and *T* stands for the matrix transposition. Both matrices *H*^*X*^ and *H*^*Z*^ have size (*n*/2) × *n*. Each row ${\bf{v}}\,\in \,{{\mathbb{F}}}_{2}^{n}$ of *H*^*X*^ defines an *X*-type check operator $X({\bf{v}})={\prod }_{j=1}^{n}{X}_{j}^{{{\bf{v}}}_{j}}$. Each row ${\bf{v}}\,\in \,{{\mathbb{F}}}_{2}^{n}$ of *H*^*Z*^ defines a *Z*-type check operator $Z({\bf{v}})={\prod }_{j=1}^{n}{Z}_{j}^{{{\bf{v}}}_{j}}$. Any *X* and *Z* checks commute as they overlap on even number of qubits (note that ${H}^{X}{({H}^{Z})}^{T}=AB+BA=0\,({\rm{mod}}\,\,2)$). By construction, the code QC(*A*, *B*) has weight-6 check operators and each qubit participates in six checks (three *X*-type plus three *Z*-type checks). Accordingly, the code QC(*A*, *B*) has a degree-6 Tanner graph. One can view the matrices *A* and *B* as bivariate polynomials over the variables *x* and *y*. Specializing BB codes to the case *m* = 1 and *B* = *A*^*T*^ gives the original bicycle codes^41^ based on univariate polynomials. Likewise, BB codes are a specialization of the generalized bicycle codes^35^, two-block group-based codes^37,42^ and polynomial-based codes^59^. Given a binary matrix *M*, let $\ker (M)$ be its nullspace spanned by all binary vectors **v** such that $M{\bf{v}}=0\,({\rm{mod}}\,\,2)$. Let rs(*M*) be the row space of *M* spanned by rows of *M*.

#### Lemma 1

The code QC(*A*, *B*) has parameters [[*n*, *k*, *d*]], where

$$\begin{array}{c}n=2{\ell }m,\,k=2\times \dim (\ker (A)\cap \ker (B))\,{\rm{a}}{\rm{n}}{\rm{d}}\,d\\ \,\,=\,\min \{|{\bf{v}}|:{\bf{v}}\in \ker ({H}^{X}){\rm{\backslash }}{\mathsf{r}}{\mathsf{s}}({H}^{Z})\}.\end{array}$$

The code offers equal distance for *X*-type and *Z*-type errors.

The proof, relying on elementary linear algebra, is deferred to the Supplementary Information. Extended Data Table 1 describes the polynomials *A* and *B* that give rise to examples of high-rate, high-distance BB codes found by a numerical search. This includes all codes from Table 1 and two examples of higher distance codes. To the best of our knowledge, all these examples are new. The code [[360, 12, ≤24]] improves on a code [[882, 24, ≤24]] with weight-6 checks found by Panteleev and Kalachev in ref. ^36^ (assuming that our distance upper bound is tight). Indeed, taking two independent copies of the 360-qubit code gives parameters [[720, 24, ≤24]]. Appendix C in ref. ^36^ also describes a code [[126, 12, 10]] that has parameters similar to ours. This code has a form QC(*A*, *B*) with *A* = 1 + *x*^43^ + *x*^37^, *B* = 1 + *x*^59^ + *x*^31^, *ℓ* = 63 and *m* = 1. We note that the recent work by Wang, Lin and Pryadko^37,38^ described examples of group-based codes closely related to the codes considered here. Some of the group-based codes with weight-8 checks found in ref. ^37^ outperform our BB codes with weight-6 checks in terms of the parameters *n*, *k*, *d*. It remains to be seen whether group-based codes can achieve a similar or better level of error suppression for the circuit-based noise model.

In the following, we partition the set of data qubits as [*n*] = *LR*, where *L* ≔ *q*(*L*) and *R* ≔ *q*(*R*) are the left and right blocks of *n*/2 = *ℓm* data qubits. Then, data qubits *L* and *R* and checks *X* and *Z* may each be labelled by integers ${{\mathbb{Z}}}_{{\ell }m}=\{0,1,\ldots ,{\ell }m-1\}$, which are indices into the matrices *A*, *B*. Alternatively, qubits and checks can be labelled by monomials from ${\mathcal{M}}=\{1,y,\ldots ,{y}^{m-1},x,xy,\ldots ,x{y}^{m-1},\ldots ,{x}^{{\ell }-1}{y}^{m-1}\}$ in this order, so that $i\in {{\mathbb{Z}}}_{{\ell }m}$ labels the same qubit or check as ${x}^{{a}_{i}}{y}^{i-m{a}_{i}}$ for *a*_*i*_ = floor(*i*/*m*). Using the monomial labelling, *L* data qubit $\alpha \in {\mathcal{M}}$ is part of *X* checks ${A}_{i}^{T}\alpha$ and *Z* checks *B*_*i*_*α* for *i* = 1, 2, 3. Similarly, *R* data qubit $\beta \in {\mathcal{M}}$ is part of *X* checks ${B}_{i}^{T}\beta$ and *Z* checks *A*_*i*_*β*. A unified notation assigns each qubit or check a label *q*(*T*, *α*) where *T* ∈ {*L*, *R*, *X*, *Z*} denotes its type and $\alpha \in {\mathcal{M}}$ its monomial label. (The monomial notations should not be confused with the matrix notations used earlier in this section. For example, multiplication of monomials such as *B*_*i*_*α* is different from multiplying a vector *α* by a matrix *B*_*i*_).

One drawback of high-rate LDPC codes is that their Tanner graphs may not be locally embeddable into the 2D grid^60,61^. This poses a challenge for hardware implementation with superconducting qubits coupled by microwave resonators. A useful very-large-scale integration (VLSI) design concept is graph thickness, see refs. ^29,62^ for details. A graph *G* = (*V*, *E*) is said to have thickness *θ* if one can partition its set of edges *E* into disjoint union of *θ* sets *E*_1_⊔*E*_2_⊔…⊔*E*_*θ*_ = *E* such that each subgraph (*V*, *E*_*i*_) is planar. Informally, a graph with thickness *θ* can be viewed as a vertical stack of *θ* planar graphs. Qubit connectivity described by a planar graph (thickness *θ* = 1) is the simplest one from hardware perspective because the couplers do not cross.

Here we show that the Tanner graph of any BB code has thickness-2. This result may be surprising as it is known that a general degree-6 graph can have thickness *θ* = 3 (ref. ^62^). Graphs with thickness *θ* = 2 might still be implementable with superconducting qubits because two planar layers of couplers and their control lines can be attached to the top and the bottom side of the chip hosting qubits.

#### Lemma 2

The Tanner graph *G* of the code QC(*A*, *B*) has thickness *θ* ≤ 2. A decomposition of *G* into two planar layers can be computed in time *O*(*n*). Each planar layer of *G* is a degree-3 graph.

#### Proof

Let *G* = (*V*, *E*) be the Tanner graph. Partition *G* into subgraphs *G*_*A*_ = (*V*, *E*_*A*_) and *G*_*B*_ = (*V*, *E*_*B*_) that describe CSS codes with check matrices

3 $$\,\mathrm{Tanner\; graph}\,\,{G}_{{\rm{A}}}:\quad {H}_{A}^{X}=[{A}_{2}+{A}_{3}| {B}_{3}]\quad {\rm{and}}\quad {H}_{A}^{Z}=[{B}_{3}^{T}| {A}_{2}^{T}+{A}_{3}^{T}]$$

4 $$\,\mathrm{Tanner\; graph}\,\,{G}_{{\rm{B}}}:\quad {H}_{B}^{X}=[{A}_{1}| {B}_{1}+{B}_{2}]\quad {\rm{and}}\quad {H}_{B}^{Z}=[{B}_{1}^{T}+{B}_{2}^{T}| {A}_{1}^{T}].$$

As *A* = *A*_1_ + *A*_2_ + *A*_3_ and *B* = *B*_1_ + *B*_2_ + *B*_3_, every edge of *G* appears either in *G*_*A*_ or *G*_*B*_, in which the two subgraphs are named by whether they contain more *A*_*i*_ edges or more *B*_*i*_ edges. Then *G*_*A*_ and *G*_*B*_ are regular degree-3 graphs (because *A*_*i*_ and *B*_*j*_ are permutation matrices).

Consider the graph *G*_*A*_. Each *X*-check vertex is connected to a pair of data vertices *i*_1_, *i*_2_ ∈ *L* by means of the matrices *A*_2_, *A*_3_ and a data vertex *i*_3_ ∈ *R* by means of the matrix *B*_3_. Each *Z*-check vertex is connected to a pair of data vertices *i*_1_, *i*_2_ ∈ *R* by means of the matrices ${A}_{2}^{T},{A}_{3}^{T}$ and a data vertex *i*_3_ ∈ *L* by means of the matrix ${B}_{3}^{T}$.

**Extended Data Fig. 1:**
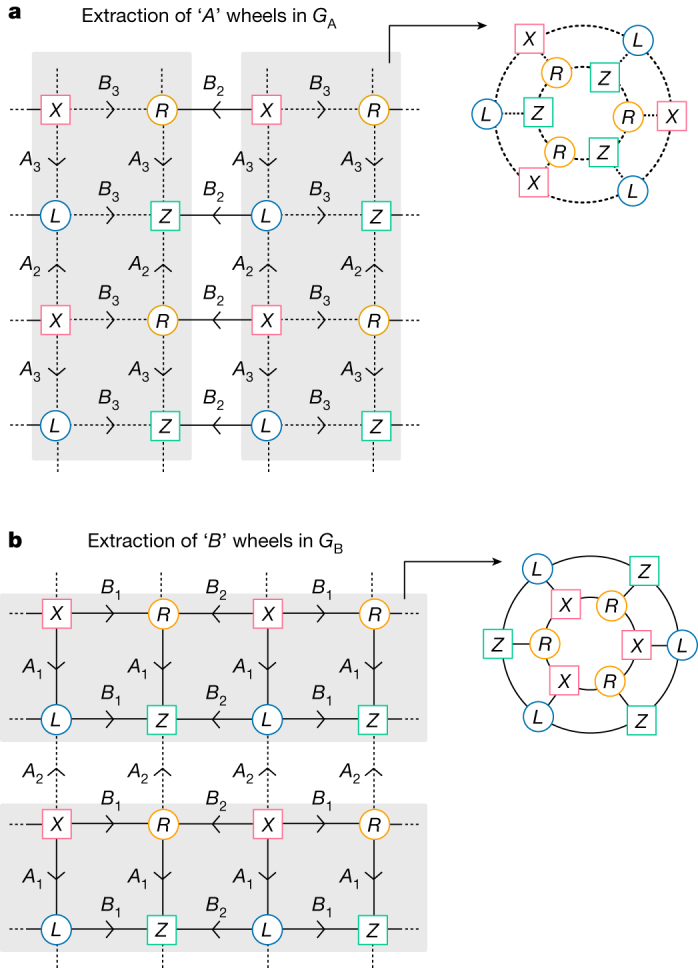
Decomposition of the Tanner graph into planar graphs. Two different grids over a torus defined using different subsets of *A*_1_, *A*_2_, *A*_3_, *B*_1_, *B*_2_, *B*_3_. Edge labels indicate adjacency matrices that generate the respective edges. By extracting either horizontal or vertical strips from these grids, we obtain planar ‘wheel graphs’ whose union contains all edges in the Tanner graph. To avoid clutter, each grid shows only a subset of edges present in the Tanner graph. **a**, The *A* wheels (dashed lines) cover *A*_2_, *A*_3_, *B*_3_. **b**, The *B* wheels (solid lines) cover *B*_1_, *B*_2_, *A*_1_.

We claim that each connected component of *G*_*A*_ can be represented by a ‘wheel graph’ illustrated in Extended Data Fig. 1. A wheel graph consists of two disjoint cycles of the same length *p* interconnected by *p* radial edges. The outer cycle alternates between *X-*check and *L* data vertices.

Edges of the outer cycle alternate between those generated by *A*_3_ (as one moves from a check to a data vertex) and ${A}_{2}^{T}$ (as one moves from a data to a check vertex). The length of the outer cycle is equal to the order of the matrix ${A}_{3}{A}_{2}^{T}$, that is, the smallest integer Ord such that ${({A}_{3}{A}_{2}^{T})}^{{\rm{O}}{\rm{r}}{\rm{d}}}={I}_{{\ell }m}$. For example, consider the code [[144, 12, 12]] from Extended Data Table 1. Then *A* = *x*^3^ + *y* + *y*^2^, *A*_2_ = *y* and *A*_3_ = *y*^2^. Thus ${A}_{3}{A}_{2}^{T}={y}^{2}{y}^{-1}=y$ that has order *m* = 6. The inner cycle of a wheel graph alternates between *Z*-check and *R*-data vertices.

Edges of the inner cycle alternate between those generated by ${A}_{3}^{T}$ (as one moves from a check to a data vertex) and *A*_2_ (as one moves from a data to a check vertex). The length of the inner cycle is equal to the order of the matrix ${A}_{3}^{T}{A}_{2}$ that is the transpose of ${A}_{3}{A}_{2}^{T}$ considered earlier. Thus both inner and outer cycles have the same length *m*. The two cycles are interconnected by *m* radial edges as shown in Extended Data Fig. 1a. Radial edges are generated by the matrix *B*_3_, as one moves towards the centre of the wheel. The wheel graph contains four cycles generated by tuples of edges $({B}_{3},{A}_{2},{B}_{3}^{T},{A}_{2}^{T})$ and $({B}_{3}^{T},{A}_{3},{B}_{3},{A}_{3}^{T})$. Commutativity between *A*_*i*_ and *B*_*j*_ ensures that traversing any of these four cycles implements the identity matrix, that is, the graph is well defined. Clearly, the wheel graph is planar. As *G*_*A*_ is a disjoint union of wheel graphs, *G*_*A*_ is planar. The same argument shows that *G*_*B*_ is planar (Extended Data Fig. 1b). The visualization of the [[144, 12, 12]] code in Fig. 1b shows the edges of *G*_*A*_ and *G*_*B*_ as dashed ‘*A*’ edges and solid ‘*B*’ edges, respectively.

We leave optimization of the code layout satisfying specific hardware constraints for future work. For now, it is sufficient to note that any planar graph admits a planar embedding without edge crossings for any prescribed vertex locations, see for example theorem 1 in ref. ^63^. Moreover, this embedding can be efficiently computed^63^. Accordingly, both planar layers in the thickness-2 decomposition of the Tanner graph can be simultaneously embedded into a plane for any fixed vertex locations such that edges do not cross within each layer.

Another example of thickness-2 graphs in the literature is the bilayer architecture of ref. ^64^. This connectivity is described by two planar graphs with additional transversal edges between them. It can be verified that bilayer graphs are thickness-2 by placing transversally connected nodes next to each other in one of the two planes and placing the transversal edges in that same plane.

The definition of code QC(*A*, *B*) does not guarantee that its Tanner graph is connected. Some choices of *A* and *B* lead to a code that is actually several separable code blocks. This manifests as a Tanner graph with several connected components. For instance, although all codes in Extended Data Table 1 are connected, taking any of them with even *ℓ* and replacing every instance of *x* with *x*^2^ creates a code with two connected components.

#### Lemma 3

The Tanner graph of the code QC(*A*, *B*) is connected if and only if $S=\{{A}_{i}{A}_{j}^{T}:i,j\in \{1,2,3\}\}\cup \{{B}_{i}{B}_{j}^{T}:i,j\in \{1,2,3\}\}$ generates the group ${\mathcal{M}}$. The number of connected components in the Tanner graph is *ℓm*/∣⟨*S*⟩∣, and all components are graph isomorphic to one another.

#### Proof

**Extended Data Fig. 2:**
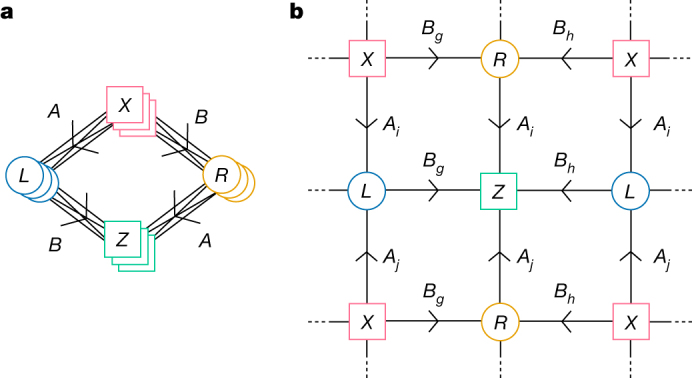
Navigating the Tanner graph. **a**, A ‘compass’ diagram that shows the direction in which matrices *A* and *B* are applied to travel between different nodes. **b**, The unit cell of the construction of a toric layout in the proof of Lemma 4.

Extended Data Fig. 2 is helpful for following the arguments in this proof. We start by proving the reverse implication of the first statement. Note that there is a length 2 path in the Tanner graph from *L* qubit $\alpha \in {\mathcal{M}}$ to *L* qubit ${A}_{i}{A}_{j}^{T}\alpha$ and another length 2 path to *L* qubit ${B}_{i}{B}_{j}^{T}\alpha$. These travel through *X* and *Z* checks, respectively. Thus, because the ${A}_{i}{A}_{j}^{T}$ and ${B}_{i}{B}_{j}^{T}$ generate ${\mathcal{M}}$, there is some path from *α* to any other *L* qubit *β*. A similar argument shows existence of a path connecting any pair of R qubits. As each *X* check and each *Z* check are connected to at least one *L* qubit and at least one *R* qubit, this implies that the entire Tanner graph is connected. The forward implication of the first statement follows after noticing that, for all *T* ∈ {*L*, *R*, *X*, *Z*}, the path from a type *T* node to any other *T* node is necessarily described as a product of elements from *S*. Connectivity of the Tanner graph implies the existence of all such paths, and so *S* must generate ${\mathcal{M}}$.

If *S* does not generate ${\mathcal{M}}$, it necessarily generates a subgroup ⟨*S*⟩ and nodes in connected components of the Tanner graph are labelled by elements of the cosets of this subgroup. This implies the theorem’s second statement.

For the next part, we establish some terminology. A spanning subgraph of a graph *G* is a subgraph containing all the vertices of *G*. Also, the undirected Cayley graph of a finite Abelian group ${\mathcal{G}}$ (with identity element 0) generated by set $S\subset {\mathcal{G}}$ is the graph with vertex set ${\mathcal{G}}$ and undirected edges (*g*, *g* + *s*) for all $g\in {\mathcal{G}}$ and all *s* ∈ *S*, *s* ≠ 0. We say the Cayley graph of ${{\mathbb{Z}}}_{a}\times {{\mathbb{Z}}}_{b}$ when we mean the Cayley graph of ${{\mathbb{Z}}}_{a}\times {{\mathbb{Z}}}_{b}$ generated by {(1, 0), (0, 1)}. The order ord(*g*) of an element *g* in a multiplicative group is the smallest positive integer such that *g*^ord(*g*)^ = 1.

#### Definition 1

Code QC(*A*, *B*) is said to have a toric layout if its Tanner graph has a spanning subgraph isomorphic to the Cayley graph of ${{\mathbb{Z}}}_{2\mu }\times {{\mathbb{Z}}}_{2\lambda }$ for some integers *μ* and *λ*.

Note that only codes with connected Tanner graphs can have a toric layout according to this definition. An example toric layout is depicted in Fig. 1b.

#### Lemma 4

A code QC(*A*, *B*) has a toric layout if there exist *i*, *j*, *g*, *h* ∈ {1, 2, 3} such that

1. $$\langle {A}_{i}{A}_{j}^{T}\,,{B}_{g}{B}_{h}^{T}\rangle ={\mathcal{M}}$$ and
2. $${\rm{ord}}({A}_{i}{A}_{j}^{T}\,){\rm{ord}}({B}_{g}{B}_{h}^{T})={\ell }m$$.

#### Proof

We let $\mu ={\rm{ord}}({A}_{i}{A}_{j}^{T}\,)$ and $\lambda ={\rm{ord}}({B}_{g}{B}_{h}^{T})$. We associate qubits and checks in the Tanner graph of QC(*A*, *B*) with elements of ${\mathcal{G}}={{\mathbb{Z}}}_{2\mu }\times {{\mathbb{Z}}}_{2\lambda }$. For *L* qubit with label $\alpha \in {\mathcal{M}}$, because of (1), there is $(a,b)\in {{\mathbb{Z}}}_{\mu }\times {{\mathbb{Z}}}_{\lambda }$ such that $\alpha ={({A}_{i}{A}_{j}^{T})}^{a}{({B}_{g}{B}_{h}^{T})}^{b}$. Because of (2) and the pigeonhole principle, this choice of (*a*, *b*) is unique. We associate *L* qubit *α* with $(2a,2b)\in {\mathcal{G}}$. Similarly, an *R* qubit with label $\alpha {A}_{j}^{T}{B}_{g}$ is associated with $(2a+1,2b+1)\in {\mathcal{G}}$, *X*-check $\alpha {A}_{j}^{T}$ with (2*a* + 1, 2*b*) and *Z*-check *αB*_*g*_ with (2*a*, 2*b* + 1). Edges in the Tanner graph ${A}_{i}\,,{A}_{j}^{T}\,,{B}_{g}$ and ${B}_{h}^{T}$ can now be drawn as in Extended Data Fig. 2b and correspond to edges in the Cayley graph of ${\mathcal{G}}$. For instance, to get from (2*a* + 1, 2*b* + 1), an *R* qubit, to (2*a* + 2, 2*b* + 1), a *Z* check, we apply *A*_*i*_, taking *R* qubit labelled $\alpha {A}_{j}^{T}{B}_{g}$ to the *Z* check labelled $(\alpha {A}_{j}^{T}{B}_{g}){A}_{i}=\alpha ({A}_{i}{A}_{j}^{T}\,){B}_{g}$.

All codes in Extended Data Table 1 have a toric layout with *μ* = *m* and *λ* = *ℓ*. Most of these codes satisfy Lemma 4 with *i* = *g* = 2 and *j* = *h* = 3. The exception is the [[90, 8, 10]] code, for which we can take *i* = 2, *g* = 1 and *j* = *h* = 3.

However, we also note two interesting cases. First, there are codes with connected Tanner graphs that do not satisfy the conditions for a toric layout given in Lemma 4. One example of such a code is QC(*A*, *B*) with *ℓ*, *m* = 28, 14, *A* = *x*^26^ + *y*^6^ + *y*^8^ and *B* = *y*^7^ + *x*^9^ + *x*^20^ that has parameters [[784, 24, ≤24]]. Second, for a code satisfying the conditions of Lemma 4, it need not be the case that the set $\{{\rm{ord}}({A}_{i}{A}_{j}^{T}\,),{\rm{ord}}({B}_{g}{B}_{h}^{T})\}$ and the set {*ℓ*, *m*} are equal. For example, the [[432, 4, ≤22]] code with *ℓ*, *m* = 18, 12 and *A* = *x* + *y*^11^ + *y*^3^, *B* = *y*^2^ + *x*^15^ + *x* only satisfies Lemma 4 with *μ*, *λ* = 36, 6 (take *i* = *g* = 1 and *j* = *h* = 2 for instance).

### Summary of other capabilities

For the remainder of this section, we summarize important details of additional capabilities of BB LDPC codes. For more details on these topics, see the Supplementary Information.

#### Syndrome circuit

Our syndrome measurement circuit relies on 2*n* physical qubits, comprising of *n* data qubits and *n* ancillary check qubits used to record the measured syndromes. It repeatedly measures the syndrome of each check operator. The single syndrome cycle is illustrated in Fig. 2. The entire syndrome measurement circuit was composed to simultaneously minimize the number of gates used, optimize depth (including parallelizing register measurement with state initialization and with gate application, whenever possible), limit the propagation of errors and comply with the qubit-to-qubit connectivity layout offered by the Tanner graph. We refer the interested reader to Supplementary Information for the complete circuit description and proof of its correctness.

We used a computer search to find a total of 936 low-depth syndrome measurement circuit alternatives. For the [[144, 12, 12]] code, the circuit shown in Fig. 2 achieves circuit distance of less than or equal to ten and we conjecture it equals ten. This syndrome measurement circuit was used to compile the data for all codes reported in Fig. 3, leaving the possibility that tailoring each of the 936 alternatives to specific codes would yield better results.

#### Decoder

We adapt the BP-OSD^36,65,66^ to the circuit noise model. This involves both an offline and online stage. In the offline stage, we take as input the syndrome measurement circuit and the error rate *p*. For every distinct single fault, we simulate the circuit efficiently using the stabilizer formalism, tracking the probability of the fault, the syndrome observed, and a final ideal syndrome. We also record in each case the logical syndrome, which indicates the logical operators anticommuting with the final error. In the online stage, we take a syndrome instance and determine a likely set of faults that occurred. Using the results of the offline stage, we can formulate this as an optimization problem, which is solved heuristically by BP-OSD.

We also leverage BP-OSD to perform two additional useful tasks that can be framed as appropriate optimization problems. First, given a code, we can find an upper bound on the code distance. Second, given a code and a syndrome measurement circuit, we can determine an upper bound on the circuit distance.

#### Logical memory capabilities

As depicted in Fig. 1c a BB LDPC code can be used as a data storage unit for, for example, a small surface code quantum computer. To this end we demonstrate two capabilities: joint logical *XX* measurements between a surface code qubit and any qubit within the BB code, and logical *Z* measurements on any qubit in the BB code. These measurements facilitate quantum teleportation circuits implementing load-store operations, transporting qubits into and out of the BB code.

These measurements are facilitated by the combination of two techniques. A construction based on ref. ^50^ enables fault-tolerant logical measurement of one logical X and one logical *Z* operator. The main idea, as illustrated in Fig. 1c, is to extend the Tanner graph of the BB code to a larger code that features the desired logical operators as stabilizers. We show that this extended Tanner graph is compatible with a thickness-2 architecture while simultaneously connecting the logical *X* extension to an ancillary surface code.

To extend the reach of these logical measurements beyond a single *X* and *Z* operator, we leverage techniques from ref. ^67^ to derive several fault-tolerant unitary operations. These operations achieve measurement of *X* and *Z* for all qubits by acting on the original measurement by conjugation, and have fault-tolerant circuit implementations within the existing connectivity of the Tanner graph.

## Online content

Any methods, additional references, Nature Portfolio reporting summaries, source data, extended data, supplementary information, acknowledgements, peer review information; details of author contributions and competing interests; and statements of data and code availability are available at 10.1038/s41586-024-07107-7.

### Supplementary information

Supplementary Information Supplementary Sections 1–5 including Supplementary Tables 1–6 and Supplementary Figs 1–3.

## Figures and Tables

**Extended Data Table 1:**
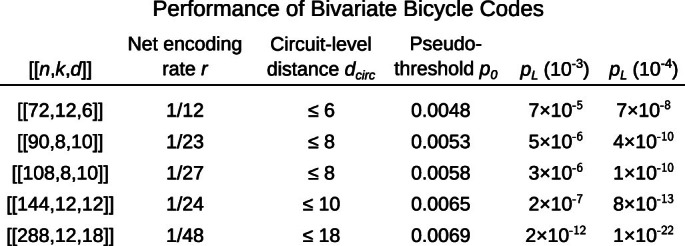
Code parameters of Bivariate Bicycle codes

Small examples of Bivariate Bicycle LDPC codes and their parameters. All codes have weight-6 checks, thickness-2 Tanner graph, and a depth-7 syndrome measurement circuit. Code distance was computed by the mixed integer programming approach of ref. ^68^. The notation ≤*d* indicates that only an upper bound on the code distance is known at the time of this writing. We round *r* down to the nearest inverse integer. The codes have check matrices *H*^*X*^ = (*A*∣*B*) and *H*^*Z*^ = (*B*^*T*^∣*A*^*T*^) with *A* and *B* defined in the last two columns. The matrices *x*, *y* obey *x*^*ℓ*^  =  *y*^*m*^  =  1 and *xy*  =  *yx*.

## Data Availability

The simulation software to generate data reported in this paper is available at https://github.com/sbravyi/BivariateBicycleCodes/.
