# Supplementary material for: High-threshold and low-overhead fault-tolerant quantum memory
Source: Nature. 2024 Mar 27;627(8005):778–82. doi: 10.1038/s41586-024-07107-7 (PMC10972743; doi:10.1038/s41586-024-07107-7)
Supplement: Supplementary file 1 — Supplementary Sections 1–5 including Supplementary Tables 1–6 and Supplementary Figs 1–3. [file 41586_2024_7107_MOESM1_ESM.pdf]

nature portfolio

https://doi.org/10.1038/s41586-024-07107-7

Supplementary information

High-threshold and low-overhead fault-tolerant quantum memory

In the format provided by the authors and unedited

Supplemental Material for
"High-threshold and low-overhead fault-tolerant quantum memory"

Sergey Bravyi[1], Andrew W. Cross[1], Jay M. Gambetta[1], Dmitri Maslov[1], Patrick Rall[2], and Theodore J. Yoder[1]

[1]IBM Quantum, IBM T.J. Watson Research Center, Yorktown Heights, NY 10598 (USA)
[2]IBM Quantum, MIT-IBM Watson AI Lab, Cambridge, MA 02142 (USA)

February 15, 2024

Contents

1 Syndrome measurement circuit 1

2 Decoder for the circuit-based noise model 5

3 Proof of Lemma 1 8

4 Numerical simulation details 10

5 Logical memory capabilities 10
- 5.1 Logical Pauli Operators . . . 12
- 5.2 Logical Gates based on Automorphisms . . . 13
- 5.3 Accessing the Primed Block via a ZX-duality . . . 15
- 5.4 Logical Measurements . . . 17

1 Syndrome measurement circuit

The next step is to furnish the code $\mathsf{QC}(A,B)$ with a syndrome measurement (SM) circuit that repeatedly measures the syndrome of each check operator. Here we describe a SM circuit that requires $2n$ physical qubits in total: n data qubits and n ancillary check qubits used to record the measured syndromes. The circuit only applies CNOTs to pairs of qubits that are connected in the Tanner graph.

The SM circuit is defined as a periodically repeated sequence of *syndrome cycles* (SC). A single SC is responsible for measuring syndromes of all n check operators of the code. Let N_c be the number of syndrome cycles. We envision that $N_c > 1$. The circuit begins and ends with a special initialization and measurement cycle responsible for initializing logical qubits in a suitable initial state and measuring logical qubits in a suitable basis. Here we focus on the optimization of the SC circuit. Logical initialization and measurements are discussed in Section 5.

The SC circuit is divided into N_r *rounds* such that each round is a depth-1 circuit composed of CNOTs and single-qubit operations. The latter include initializing a qubit in the X or Z basis and measuring a qubit in the X or Z basis. CNOTs can be applied only to pairs of qubits which are nearest neighbors in the Tanner graph. Some qubits remain idle during some rounds, although we try to minimize such occurrences by squeezing more useful computations in as little time as possible. Our notations are summarized in Table 1.

Notation	Operation
CNOT c t	CNOT with control qubit c and target qubit t
InitX q	Initialize qubit q in the state $\|+\rangle = (\|0\rangle + \|1\rangle)/\sqrt{2}$
InitZ q	Initialize qubit q in the state $\|0\rangle$
MeasX q	Measure qubit q in the X-basis $\|+\rangle, \|-\rangle$
MeasZ q	Measure qubit q in the Z-basis $\|0\rangle, \|1\rangle$
Idle q	Identity gate on qubit q

Table 1: Elementary operations used for syndrome measurements.

Below we describe a SC circuit with effectively $N_r = 8$ rounds[1]. Ignoring single-qubit initialization and measurement operations, the SC circuit is a depth-7 CNOT circuit. By designing the circuit for an explicit family of LDPC codes we are able to leverage the symmetries and reduce computational depth to 7 from what otherwise would be $14 = 2\cdot 6 + 2$, as shown by previous authors [1, Theorem 1]. Our notations are as follows. We divide n data qubits into the left and the right registers $q(L)$ and $q(R)$ of size $n/2$ each. Each check operator acts on three data qubits from $q(L)$ and three data qubits from $q(R)$. The SM circuit uses $2n$ physical qubits in total: n data qubits and n ancillary check qubits that record the syndrome of each check operator. Let $q(X)$ and $q(Z)$ be the ancillary registers of size $n/2$ that span X-check and Z-check qubits respectively. Thus the physical qubits are partitioned into four registers, $q(X)$, $q(L)$, $q(R)$, and $q(Z)$, of size $n/2$ each. Label qubits in each register by integers $i = 1, 2, \ldots, n/2$. We write $q(X, i)$ for the i-th qubit of the register $q(X)$ with similar notations for $q(L)$, $q(R)$, and $q(Z)$. Each permutation matrix A_p and B_q from Eq. (1) in the main text defines a one-to-one map from the set $\{1, 2, \ldots, n/2\}$ onto itself.

We identify a permutation matrix and the corresponding one-to-one map. For example, we write $j = A_1(i)$ if the matrix A_1 has a one at row i and column j (this is well defined since A_1 is a permutation matrix). Likewise, we write $j = A_1^T(i)$ if the transposed matrix A_1^T has a one at the row i and column j. In this notation, the i-th X-check operator acts on data qubits $q(L, A_p(i))$ and $q(R, B_p(i))$ with $p = 1, 2, 3$. The i-th Z-check operator acts on data qubits $q(L, B_p^T(i))$ and $q(R, A_p^T(i))$ with $p = 1, 2, 3$.

Our depth-8 SC circuit is described in Table 2 and illustrated in Figure 2 in the main text. Note that within each round all operations act over non-overlapping sets of qubits. In particular, each round applies at most one layer of CNOT gates between $q(X)$ and $q(L)$ registers (Rounds 2, 6, and 7), at most one layer of CNOTs between $q(X)$ and $q(R)$ registers (Rounds 3, 4, and 5), at most one layer of CNOTs between $q(Z)$ and $q(L)$ registers (Rounds 3, 4, and 5), and at most one layer of CNOTs between $q(Z)$ and $q(R)$ registers (Rounds 1, 2, and 6). Qubits from $q(Z)$ are always targets for CNOTs. Accordingly, X-type errors propagate from data qubits to check qubits in $q(Z)$. The latter are measured in the Z-basis in Round 7 revealing the syndrome of X-type errors. Qubits from $q(X)$ are always controls for CNOTs. Accordingly, Z-type errors propagate from data qubits to check qubits in $q(X)$. The latter are measured in the X-basis in Round 8 revealing the syndrome of Z-type errors. We envision that the syndrome cycles are repeated periodically. This justifies applying CNOTs to $q(Z)$ at Round 1 even though $q(Z)$ is initialized only at Round 8. Indeed, Round 8 of the previous syndrome cycle goes immediately before Round 1 of the current cycle. Thus $q(Z)$ has been already initialized at the beginning of Round 1. We were not able to find a depth-8 (or smaller depth) syndrome cycle in which X-check and Z-check qubits are initialized and measured synchronously.

Let us now prove that the above SC circuit has the desired functionality. Since the circuit involves only Clifford operations, its action can be compactly described using stabilizer tableau [2]. We track how the tableau changes as each layer of CNOTs in the circuit is applied. Since the CNOT gates do not mix Pauli X and Z operators, one may consider tableau describing the action of the circuit on X-type and Z-type Pauli operators separately.

Let us begin with X-type Pauli. The corresponding tableau T is a binary matrix of size $n \times 2n$ such that each row of T defines an X-type stabilizer of the underlying quantum state. We partition columns of T into four blocks that represent qubit registers $q(X)$, $q(L)$, $q(R)$, and $q(Z)$. We partition rows of T into two blocks such that initially the top $n/2$ rows represent weight-1 check operators on qubits of the register $q(X)$ initialized in the state $|+\rangle$ while the bottom $n/2$ rows represent weight-6 check operators on data qubits associated with the chosen code $\mathsf{QC}(A, B)$. Thus,

[1]The operator InitZ $q(Z, i)$ must be executed before the first application of this SC circuit, raising the depth of the first stage to 9. However, each following syndrome cycle takes Z-check initialization from the previous round. Last syndrome cycle needs not apply the Z-check state initialization. Total SM circuit depth with N_c syndrome cycles is thus $8N_c + 1$.

Round	Circuit	Round	Circuit
1	**for** $i = 1$ to $n/2$ **do**<br>InitX $q(X, i)$<br>CNOT $q(R, A_1^T(i))$ $q(Z, i)$<br>Idle $q(L, i)$<br>**end for**	5	**for** $i = 1$ to $n/2$ **do**<br>CNOT $q(X, i)$ $q(R, B_3(i))$<br>CNOT $q(L, B_3^T(i))$ $q(Z, i)$<br>**end for**
2	**for** $i = 1$ to $n/2$ **do**<br>CNOT $q(X, i)$ $q(L, A_2(i))$<br>CNOT $q(R, A_3^T(i))$ $q(Z, i)$<br>**end for**	6	**for** $i = 1$ to $n/2$ **do**<br>CNOT $q(X, i)$ $q(L, A_1(i))$<br>CNOT $q(R, A_2^T(i))$ $q(Z, i)$<br>**end for**
3	**for** $i = 1$ to $n/2$ **do**<br>CNOT $q(X, i)$ $q(R, B_2(i))$<br>CNOT $q(L, B_1^T(i))$ $q(Z, i)$<br>**end for**	7	**for** $i = 1$ to $n/2$ **do**<br>CNOT $q(X, i)$ $q(L, A_3(i))$<br>MeasZ $q(Z, i)$<br>Idle $q(R, i)$<br>**end for**
4	**for** $i = 1$ to $n/2$ **do**<br>CNOT $q(X, i)$ $q(R, B_1(i))$<br>CNOT $q(L, B_2^T(i))$ $q(Z, i)$<br>**end for**	8	**for** $i = 1$ to $n/2$ **do**<br>MeasX $q(X, i)$<br>InitZ $q(Z, i)$<br>Idle $q(L, i)$<br>Idle $q(R, i)$<br>**end for**

Table 2: Depth-8 syndrome measurement cycle circuit.

at the beginning of Round 1, when all check qubits in the register $q(X)$ have been initialized in the state $|+\rangle$, while data qubits are in some logical state of the code $\mathsf{QC}(A, B)$, the binary matrix is

$$\begin{pmatrix} I & 0 & 0 & 0 \\ 0 & A & B & 0 \end{pmatrix}.$$

Here $I \equiv I_{n/2}$ is the identity matrix. The SC circuit (ignoring qubit initialization and measurements) enacts the transformation

$$\begin{pmatrix} I & 0 & 0 & 0 \\ 0 & A & B & 0 \end{pmatrix} \xrightarrow{\text{SC circuit}} \begin{pmatrix} I & A & B & 0 \\ 0 & A & B & 0 \end{pmatrix}.$$

Indeed, the circuit must map a single-qubit X stabilizer X_j on a check qubit $j \in q(X)$ to a product of X_j and the j-th X-type check operator on the data qubits determined by the j-th row of $H^X = [A|B]$. The eigenvalue measurement of X_j at the final round then reveals the syndrome of the j-th check operator. The bottom $n/2$ rows must be unchanged since the check operators of the code must be the same before and after the syndrome measurement.

Let us verify that the circuit defined in Table 2 enacts the desired transformation. To accomplish this, we rewrite the SC circuit by removing notations irrelevant to showing the correctness of X-checks. Specifically, we write each CNOT in Table 2 as $\mathsf{CNOT}_M(a, b)$, where $a, b \in \{1, 2, 3, 4\} = \{q(X), q(L), q(R), q(Z)\}$, and $M \in \{A_1, A_2, A_3, B_1, B_2, B_3\}$. Note that the CNOT instructions where the matrix M^T is used instead of M can be written using matrix M by performing the variable renaming $i \leftarrow M(i)$ in the corresponding for loop in Table 2.

Using the above compact notation, the unitary part of the SC circuit becomes:

$$\begin{array}{ll} \text{Round 1:} & \mathsf{CNOT}_{A_1}(3,4) \\ \text{Round 2:} & \mathsf{CNOT}_{A_2}(1,2), \mathsf{CNOT}_{A_3}(3,4) \\ \text{Round 3:} & \mathsf{CNOT}_{B_2}(1,3), \mathsf{CNOT}_{B_1}(2,4) \\ \text{Round 4:} & \mathsf{CNOT}_{B_1}(1,3), \mathsf{CNOT}_{B_2}(2,4) \\ \text{Round 5:} & \mathsf{CNOT}_{B_3}(1,3), \mathsf{CNOT}_{B_3}(2,4) \\ \text{Round 6:} & \mathsf{CNOT}_{A_1}(1,2), \mathsf{CNOT}_{A_2}(3,4) \\ \text{Round 7:} & \mathsf{CNOT}_{A_3}(1,2) \end{array} \quad (1)$$

In the following we apply all seven unitary rounds to verify the correctness of the performed transformation:

$$\text{Round 1:} \begin{pmatrix} I & 0 & 0 & 0 \\ 0 & A & B & 0 \end{pmatrix} \xrightarrow{\mathsf{CNOT}_{A_1}(3,4)} \begin{pmatrix} I & 0 & 0 & 0 \\ 0 & A & B & A_1B \end{pmatrix}$$

$$\text{Round 2:} \xrightarrow{\mathsf{CNOT}_{A_2}(1,2)} \begin{pmatrix} I & A_2 & 0 & 0 \\ 0 & A & B & A_1B \end{pmatrix} \xrightarrow{\mathsf{CNOT}_{A_3}(3,4)} \begin{pmatrix} I & A_2 & 0 & 0 \\ 0 & A & B & (A_1{+}A_3)B \end{pmatrix}$$

$$\text{Round 3:} \xrightarrow{\mathsf{CNOT}_{B_2}(1,3)} \begin{pmatrix} I & A_2 & B_2 & 0 \\ 0 & A & B & (A_1{+}A_3)B \end{pmatrix} \xrightarrow{\mathsf{CNOT}_{B_1}(2,4)} \begin{pmatrix} I & A_2 & B_2 & A_2B_1 \\ 0 & A & B & (A_1{+}A_3)B + AB_1 \end{pmatrix}$$

$$\text{Round 4:} \xrightarrow{\mathsf{CNOT}_{B_1}(1,3)} \begin{pmatrix} I & A_2 & B_1{+}B_2 & A_2B_1 \\ 0 & A & B & (A_1{+}A_3)B + AB_1 \end{pmatrix}$$

$$\xrightarrow{\mathsf{CNOT}_{B_2}(2,4)} \begin{pmatrix} I & A_2 & B_1{+}B_2 & A_2(B_1{+}B_2) \\ 0 & A & B & (A_1{+}A_3)B + A(B_1{+}B_2) \end{pmatrix} = \begin{pmatrix} I & A_2 & B_1{+}B_2 & A_2(B_1{+}B_2) \\ 0 & A & B & A_2B + AB_3 \end{pmatrix}$$

Here, we use the identity $(A_1{+}A_3)B + A(B_1{+}B_2) = A_2B + AB_3$, which holds since the sum of first summands and second summands on both sides of the equation gives AB, and $AB + AB = 0$.

$$\text{Round 5:} \xrightarrow{\mathsf{CNOT}_{B_3}(1,3)} \begin{pmatrix} I & A_2 & B & A_2(B_1{+}B_2) \\ 0 & A & B & A_2B + AB_3 \end{pmatrix} \xrightarrow{\mathsf{CNOT}_{B_3}(2,4)} \begin{pmatrix} I & A_2 & B & A_2B \\ 0 & A & B & A_2B \end{pmatrix}$$

$$\text{Round 6:} \xrightarrow{\mathsf{CNOT}_{A_1}(1,2)} \begin{pmatrix} I & A_1{+}A_2 & B & A_2B \\ 0 & A & B & A_2B \end{pmatrix} \xrightarrow{\mathsf{CNOT}_{A_2}(3,4)} \begin{pmatrix} I & A_1{+}A_2 & B & 0 \\ 0 & A & B & 0 \end{pmatrix}$$

$$\text{Round 7:} \xrightarrow{\mathsf{CNOT}_{A_3}(1,2)} \begin{pmatrix} I & A & B & 0 \\ 0 & A & B & 0 \end{pmatrix}.$$

This is the desired transformation.

So far, we have not considered the action of the SC circuit on the logical qubits of the code. Let us show that this action is trivial. Indeed, consider some X-type logical operator $X(v)$, where $v \in \mathbb{F}_2^n$. Write $v = (u, w)$ where u and w are restrictions of v onto the registers 2 and 3 respectively. Commutativity between $X(v)$ and any Z-type check operator implies

$$uB + wA = 0.$$

Here we consider u and w as row vectors. Extending v by zeroes on registers 1 and 4 gives the row vector $(0\ u\ w\ 0)$, where 0 stands for the all-zero row vector of length $n/2$. Let us follow the same chain of transformations as above starting from the initial vector $(0\ u\ w\ 0)$. All CNOTs controlled by the register 1, such as $\mathsf{CNOT}_{A_2}(1,2)$ or $\mathsf{CNOT}_{B_2}(1,3)$ in Eq. (1), have trivial action on the vector $(0\ u\ w\ 0)$ since all qubits of the control register are zeroes. Such CNOTs can be omitted. The remaining CNOTs in Eq. (1) such as $\mathsf{CNOT}_{A_1}(3,4)$ or $\mathsf{CNOT}_{B_1}(2,4)$ map the initial vector $(0\ u\ w\ 0)$ to $(0\ u\ w\ t)$ for some vector t since the registers 2 and 3 always serve as the controls and the register 4 always serves as the target. Rounds 1, 2, and 6 in Eq. (1) are equivalent to XORing vectors wA_1, wA_3, and wA_2 respectively to the register 4. Rounds 3, 4, and 5 in Eq. (1) are equivalent to XORing vectors uB_1, uB_2, and uB_3 respectively to the register 4. Thus

$$t = w(A_1{+}A_2{+}A_3) + u(B_1{+}B_2{+}B_3) = wA + uB = 0.$$

We have shown that the SC circuit maps the vector $(0\ u\ w\ 0)$ to itself. Hence the circuit acts trivially on logical X-type operators.

To prove the correctness of Z-checks, observe that Z-checks can be mapped into X-checks by conjugation with Hadamards. When the unitary circuit in Figure 2 in the main text is conjugated with Hadamards, this flips controls and targets of all CNOT gates. Thus, to verify Z-checks, it suffices to perform a very similar calculation to the one already shown for X-checks. We omit this calculation here.

A key metric to evaluate a SM circuit is what we call the circuit-level distance d_{circ}. A SM circuit has circuit-level distance d_{circ} if it takes at least d_{circ} faulty operations in the circuit to generate a logical error without triggering any syndromes. By definition, $d_{\mathsf{circ}} \le d$ for any distance-d code and typically $d_{\mathsf{circ}} < d$ since the SM circuit can convert a single-qubit error to a high-weight error spanning many qubits. We say that a SM circuit is distance-preserving if $d_{\mathsf{circ}} = d$, which is the best one can hope for. Numerical simulations suggest that our SM circuit is nearly distance-preserving such that d_{circ} is close to d for all considered codes and $d_{\mathsf{circ}} = d$ for some codes, see Table 1 in the main text. Details of calculating upper bounds on the circuit-level distance are provided in Section 2.

The SC circuit shown in Table 2 is not unique in the following sense: we found 935 depth-7 alternatives to the unitary part of the SC circuit via a computer search. These alternatives are obtained from the circuit defined in Eq. (1) by applying the gate layers CNOT_{A_i} and CNOT_{B_j} in a different order. In the special case of the $[[144, 12, 12]]$ code, numerical simulations show that all 936 variants of the syndrome cycle give rise to syndrome measurement circuits with distance $d_{\mathsf{circ}} \le 10$ explaining our focus on a specific circuit Eq. (1) which we conjecture to have distance $d_{\mathsf{circ}} = 10$. The short depth of the single cycle, relying on only seven computational stages, helps to keep the spread of errors under control.

2 Decoder for the circuit-based noise model

So far we assumed that the SM circuit is noiseless. As shown in Section 1, in this case all measured syndromes are zero and the circuit implements the logical identity gate. Consider now what happens when each operation in the circuit including CNOT gates, qubit initializations, measurements, and idle qubits is subject to noise. To enable efficient decoding and numerical simulations, we use the standard circuit-based depolarizing noise model [3]. It assumes that each operation in the circuit is ideal or faulty with the probability $1-p$ or p respectively. Here p is a model parameter called the error rate. Faults on different operations occur independently. We define faulty operations as follows. A faulty CNOT is an ideal CNOT followed by one of 15 non-identity Pauli errors on the control and the target qubits picked uniformly at random. A faulty initialization prepares a single-qubit state orthogonal to the correct one. A faulty measurement is an ideal measurement followed by a classical bit-flip error applied to the measurement outcome. A faulty idle qubit suffers from a Pauli error X or Y or Z picked uniformly at random.

To perform error correction one needs a decoder — a classical algorithm that takes as input the measured error syndrome and outputs a guess of the final Pauli error on the data qubits resulting from all faults in the SM circuit. The error syndrome may itself be faulty due to measurement errors. The decoder succeeds if the guessed Pauli error coincides with the actual error up to a product of check operators. In this case the guessed and the actual error have the same action on any logical state.

Let us show how to adapt Belief Propagation with an Ordered Statistics postprocessing step Decoder (BP-OSD) proposed in [4, 5] to the circuit-based noise model. The decoder consists of two stages. The first stage takes as input a BB code $\mathsf{QC}(A, B)$ equipped with a SM circuit $\mathcal{U}$ and an error rate p. It outputs a certain linearized noise model that ignores possible cancellations between errors generated by two or more faulty operations in $\mathcal{U}$. This stage is analogous to computing the decoding graph in error correction algorithms based on the surface code [6, 7]. The second (online) stage of the decoder takes as input an error syndrome measured in the experiment and outputs a guess of the final error on the data qubits. This stage decodes the linearized noise model using BP-OSD method [4, 5]. Our linearized noise model is conceptually similar to spacetime codes studied by Delfosse and Paetznick [8] and detector-based noise model proposed by McEwen, Bacon, and Gidney [9]. The online stage of our decoder closely follows Refs. [10, 11]. In particular, Gehér, Crawford, and Campbell [11] applied BP-OSD to study tangled syndrome measurement circuits capable of measuring certain non-local check operators on a hardware with short-range qubit connectivity. Higgott et al [10] showed that the performance of the standard minimum-weight matching decoder can be enhanced by computing prior error probabilities using BP-decoder as a preprocessing step.

We begin by describing the offline stage. Consider a BB code with parameters $[[n, k, d]]$ and let $\mathcal{U}$ be the SM

circuit constructed in Section 1 with N_c syndrome cycles. The circuit $\mathcal{U}$ contains $6nN_c$ CNOTs, nN_c initializations and measurements, and $2nN_c$ idle qubit locations. Let $\mathcal{U}_1, \mathcal{U}_2, \ldots, \mathcal{U}_M$ be the list of all possible faulty realizations of $\mathcal{U}$ with exactly one faulty operation. If the faulty operation happens to be CNOT or an idle qubit, one of the admissible Pauli errors for this operation is specified. A simple counting shows that $M = 98nN_c$, where $98 = 15 \cdot 6 + 1 + 1 + 3 \cdot 2$ accounts for 15 noisy realizations of each CNOT, 3 realizations of memory errors on idle qubits, noisy initializations and measurements. By definition, the list $\mathcal{U}_1, \mathcal{U}_2, \ldots, \mathcal{U}_M$ includes all realizations of $\mathcal{U}$ that can occur with the probability $O(p)$ in the limit $p \to 0$. We simulate each circuit $\mathcal{U}_j$ by propagating the chosen Pauli error towards the final time step taking into account qubit initialization and measurement errors (if any). This simulation can be performed efficiently using the stabilizer formalism. Let $s_j^U \in \{0,1\}^{nN_c}$ be the full measured syndrome of $\mathcal{U}_j$ and E_j be the final n-qubit Pauli error on the data qubits generated by $\mathcal{U}_j$. Let $s_j^F \in \{0,1\}^n$ be the syndrome of the final error E_j. In other words, if we write $E_j = X(\alpha_j)Z(\beta_j)$ for some vectors $\alpha_j, \beta_j \in \{0,1\}^n$, then

$$s_j^F = \left[\begin{array}{c} H^Z \alpha_j \\ H^X \beta_j \end{array} \right].$$

Here H^X and H^Z are the check matrices of the chosen code. Finally, let $s_j^L \in \{0,1\}^{2k}$ be a *logical syndrome* of the final error E_j defined as follows. Fix some basis set of logical Pauli operators $\overline{P}_1, \overline{P}_2, \ldots, \overline{P}_{2k}$ for the chosen code. For example, $\overline{P}_1, \overline{P}_2, \ldots, \overline{P}_k$ could be logical X-type operators and $\overline{P}_{k+1}, \overline{P}_{k+2}, \ldots, \overline{P}_{2k}$ could be logical Z-type operators. The i-th bit of s_j^L is defined as

$$(s_j^L)_i = \begin{cases} 1 & \text{if} \quad E_j \overline{P}_i = -\overline{P}_i E_j, \\ 0 & \text{if} \quad E_j \overline{P}_i = \overline{P}_i E_j, \end{cases}$$

for $i = 1, \ldots, 2k$. Note that the pair of syndromes s_j^F, s_j^L uniquely determines the final error E_j modulo check operators. Define a pair of *decoding matrices* D and D^L of size $(nN_c + n) \times M$ and $2k \times M$ respectively such that the j-th column of D is

$$\left[\begin{array}{c} s_j^U \\ s_j^F \end{array} \right]$$

and the j-th column of D^L is s_j^L. Let p_j be the probability of a Pauli error that occurred in the circuit $\mathcal{U}_j$. We have $p_j = p/15$ if $\mathcal{U}_j$ contains a faulty CNOT, $p_j = p/3$ if $\mathcal{U}_j$ contains a faulty idle qubit, and $p_j = p$ if $\mathcal{U}_j$ contains a faulty qubit initialization or measurement. Suppose $I \subseteq \{1, 2, \ldots, M\}$ is a subset of columns of D such that triples of syndromes (s_j^U, s_j^F, s_j^L) are the same for all $j \in I$. We merge all columns in I to a single column and assign the value $\sum_{j \in I} p_j$ to the bit-flip error probability associated with the merged column. Let M be the number of columns of D after the merging step and $p_1, p_2, \ldots, p_M$ be the respective error probabilities.

Next, the decoding matrix D is converted to a sparse form. To this end consider a faulty circuit $\mathcal{U}_j$ and a sequence of syndromes measured by $\mathcal{U}_j$ on some check operator. Let this sequence be $m = (m_1, m_2, \ldots, m_{N_c}) \in \{0,1\}^{N_c}$. Since $\mathcal{U}_j$ contains a single fault, the sequence m has only a few locations where the measured syndromes differ at two consecutive cycles. For example, if $\mathcal{U}_j$ contains a Pauli error on some idle data qubit between two syndrome cycles, the m-sequence may look as $(0, 0, \ldots, 0, 1, 1, \ldots, 1)$. Such sequence can be made sparse if we represent it by a binary vector

$$m' = (m_1, m_2 \oplus m_1, m_3 \oplus m_2, \ldots, m_{N_c} \oplus m_{N_c - 1}) \in \{0,1\}^{N_c}.$$

In other words, m' stores changes in the measured syndrome at a given check operators at each cycle. We convert the matrix D to a sparse form by applying the map $m \to m'$ to the syndromes measured by each check operator for each faulty circuit $\mathcal{U}_j$.

Let $\xi_1, \xi_2, \ldots, \xi_M \in \{0,1\}$ be independent random variables such that ξ_j takes values 0 and 1 with the probability $1 - p_j$ and p_j respectively. Define a linearized noise model that outputs a random triple (s^U, s^F, E), where

$$E = \prod_{j=1}^{M} (E_j)^{\xi_j}$$

is an n-qubit Pauli error and

$$\left[\begin{array}{c} s^U \\ s^F \end{array} \right] = \sum_{j=1}^{M} \xi_j \left[\begin{array}{c} s_j^U \\ s_j^F \end{array} \right] \pmod 2$$

is a binary vector that represents the error syndrome. The linearized model is a simplified version of the circuit-based noise that ignores possible cancellations among errors generated by two or more faulty operations in $\mathcal{U}$. Note that such errors occur with the probability only $O(p^2)$. The decoder attempts to guess the final error E acting on the data qubits based on the syndrome s^U measured in the experiment making a simplifying assumption that that the pair (s^U, E) was generated using the linearized noise model. We additionally assume that the decoder knows the syndrome s^F of the final error E. This syndrome can be acquired by adding one noiseless cycle at the end of the syndrome measurement circuit, which is a common practice in numerical simulations of error correction. By definition, we have

$$D\xi = \left[\begin{array}{c} s^U \\ s^F \end{array}\right].$$

Here $\xi = (\xi_1, \xi_2, \ldots, \xi_M)$ is a column vector and matrix-vector multiplication is modulo two. Define a minimum weight error $\xi^* = \xi^*(s) \in \{0,1\}^M$ as a solution of an optimization problem

$$\xi^* = \arg \min_{\xi \in \{0,1\}^M} \sum_{j=1}^{M} \log{(1/p_j)} \xi_j \quad \text{subject to} \quad D\xi = \left[\begin{array}{c} s^U \\ s^F \end{array}\right]. \tag{2}$$

This problem is equivalent to the minimum weight decoding for a length-M linear code with the check matrix D, bit-flip error probabilities $p_1, p_2, \ldots, p_M$, and noiseless syndromes. Our guess of the unknown logical syndrome is

$$s^L = D^L \xi^*.$$

Let E^* be any n-qubit Pauli operator with the syndrome s^F and the logical syndrome s^L. Note that E^* is defined uniquely modulo multiplication by check operators. The Pauli E^* is our guess of the final error on the data qubits. Let E be the actual final error on the data qubits generated by a noisy realization of $\mathcal{U}$ without making any simplifications of the noise model. By definition, Pauli operators E and E^* have the same syndrome but they may differ by a logical Pauli operator. We declare a logical error if E and E^* differ by any non-identity logical operator (there are $4^k - 1$ choices of this logical operator). Otherwise the decoding is deemed successful.

It remains to explain how to solve the optimization problem Eq. (2). Since the minimum weight decoding for a linear code is known to be NP-hard problem [12], finding the exact solution of Eq. (2) might be practically impossible for problem instances with several thousand variables that we have to deal with. Furthermore, estimation of the logical error probability p_L by the Monte Carlo method requires solving $O(1/p_L)$ instances of the problem Eq. (2). This number can be quite large since p_L is a small parameter. To address these challenges, we employ the BP-OSD algorithm [4, 5]. Recall that belief propagation (BP) is a heuristic message passing algorithm aimed at computing single-bit marginals of a probability distribution

$$P(\xi|\sigma) = \left\{ \begin{array}{cl} \frac{1}{\mathcal{Z}} \prod_{j=1}^{M} (1-p_j)^{1-\xi_j} p_j^{\xi_j} & \text{if} \quad D\xi = \sigma, \\ 0 & \text{otherwise.} \end{array}\right.$$

Here $\xi \in \{0,1\}^M$ and $\mathcal{Z}$ is a normalization factor chosen such that $\sum_{\xi \in \{0,1\}^M} P(\xi|\sigma) = 1$. In our case ξ represents an unknown error in the linearized noise model, D is the decoding matrix constructed above, and $\sigma = \left[\begin{array}{c} s^U \\ s^F \end{array}\right]$ is the measured error syndrome. Let $q_j \in [0,1]$ be an estimate of the marginal probability $\Pr[\xi_j = 1]$ obtained by the belief propagation method with some fixed number of message passing iterations. The ordered statistics post-processing step examines *information sets* which are subsets of bits $I \subseteq [M]$ such that the linear system $D\xi = \sigma$ has a unique solution ξ supported on I, that is, $\xi_j = 0$ for all $j \notin I$. Information sets are ranked according to their *reliability* which is defined as

$$\rho(I) = \prod_{j \in I} \max{(q_j, 1-q_j)}.$$

BP-OSD finds an information set I with the largest reliability using a greedy algorithm [4]. The final output of BP-OSD is a solution of the system $D\xi = \sigma$ supported on the most reliable information set I. We replace the minimum weight error ξ^* in Eq. (2) by the solution ξ proposed by BP-OSD.

Since BB LDPC codes are of CSS-type, it is natural to decode X-type and Z-type errors independently. Accordingly, we solve the minimum weight decoding problem Eq. (2) twice with a pair of decoding matrices D_X and

D_Z constructed as above but including only the syndromes of X-type and Z-type check operators respectively. This results in guessed X-type and Z-type errors E^*_X and E^*_Z. The guessed final error is $E^* = E^*_X E^*_Z$. We empirically observed that the resulting decoding matrices D_X and D_Z are $(6, 35)$-sparse for any BB code, meaning that there are at most 6 nonzeros in each column and at most 35 nonzeros in each row of D_X and D_Z. The number of columns scales as $O(nN_c)$ where the constant coefficient depends on a particular code. For example, decoding matrices D_X and D_Z describing the code $[[144, 12, 12]]$ with $N_c = 12$ syndrome cycles have 8857 and 8785 columns respectively.

We also employed BP-OSD to compute an upper bound on the code distance d. Consider a CSS-type LDPC code $[[n, k, d]]$ with check matrices H^X and H^Z. Assume for simplicity that this code has the same distance for X- and Z-type errors (this assumption is satisfied for BB LDPC codes due to Lemma 1 in the main text). Suppose $Z(\xi)$ is a minimum weight logical Z-type operator. Then $\xi \in \mathsf{ker}(H^X)$ and $\xi \notin \mathsf{rs}(H^Z)$. Let $X(\eta)$ be any logical X-type operator. Here $\eta \in \mathsf{ker}(H^Z) \setminus \mathsf{rs}(H^X)$. Consider the following optimization problem:

$$d(\eta) = \min_{\xi \in \mathsf{ker}(H^X)} \sum_{j=1}^{n} \xi_j \quad \text{subject to} \quad \eta^T \xi = 1. \tag{3}$$

Then $d(\eta) \geq d$ for any logical operator $X(\eta)$ and $d(\eta) = d$ if $X(\eta)$ anti-commutes with some minimum-weight logical operator $Z(\xi)$. The latter event occurs with the probability $1/2$ if one picks $\eta \in \mathsf{ker}(H^Z)$ uniformly at random. In this case $d(\eta) = d$ with the probability at least $1/2$ and $d(\eta) \geq d$ with certainty. Let $d^{\mathsf{BP}}(\eta)$ be an upper bound on $d(\eta)$ obtained by solving the optimization problem Eq. (3) using BP-OSD method with a parity check matrix $\begin{bmatrix} H^X \\ \eta^T \end{bmatrix}$ and a syndrome $(0, 0, \ldots, 0, 1)^T$. We have $d^{\mathsf{BP}}(\eta) \geq d$ with certainty and $d^{\mathsf{BP}}(\eta) = d$ with the probability $1/2$ whenever BP-OSD finds the optimal solution. Choose the number trials $T \gg 1$ and pick vectors $\eta^1, \eta^2, \ldots, \eta^T \in \mathsf{ker}(H^Z) \setminus \mathsf{rs}(H^X)$ uniformly at random. Then

$$d^{\mathsf{BP}} := \min_{a=1,2,\ldots,T} d^{\mathsf{BP}}(\eta^a)$$

is an upper bound on the distance d that can be systematically improved by increasing the number of trials T.

Using the quantity d^{BP} as an efficiently computable proxy for the code distance enabled us to search over a large number of candidate BB codes with $n = O(100)$ qubits. The vast majority of these candidates was discarded due to an insufficiently large upper bound d^{BP}. This left only a few viable candidates with a sufficiently large value of d^{BP}. The actual distance of each candidate code was computed using the integer linear programming method [13].

We similarly computed an upper bound on the circuit-level distance d_{circ}. Since the SM circuit can break the symmetry between X- and Z-type errors, the circuit-level distance has to be computed for both types of errors. For concreteness, let us discuss the circuit-level distance d^Z_{circ} for Z-type errors. The latter is defined as the minimum number of faulty operations in the SM circuit that can generate an undetectable Z-type logical error. The optimization problem Eq. (3) is replaced by

$$d^Z_{\mathsf{circ}}(\eta) = \min_{\xi \in \mathsf{ker}(D_X)} \sum_{j=1}^{M} \xi_j \quad \text{subject to} \quad \eta^T \xi = 1, \tag{4}$$

where D_X is the decoding matrix constructed above and $\eta \in \{0, 1\}^M$ is a random linear combination of rows of D_X and rows of D_L that represent logical X-type operators. Then $d_{\mathsf{circ}}(\eta) \geq d^Z_{\mathsf{circ}}$ with certainty and $d_{\mathsf{circ}}(\eta) = d^Z_{\mathsf{circ}}$ with the probability at least $1/2$. Solving the optimization problem Eq. (4) using BP-OSD method for many random choices of the vector η and taking the minimum value of $d^Z_{\mathsf{circ}}(\eta)$ provides an upper bound on d^Z_{circ}. One can similarly compute an upper bound on the circuit-level distance d^X_{circ} for X-type errors. This provides an upper bound on $d_{\mathsf{circ}} = \min(d^X_{\mathsf{circ}}, d^Z_{\mathsf{circ}})$.

3 Proof of Lemma 1

For convenience of the reader we restate the lemma below.

Lemma 1. *The code* $\mathsf{QC}(A, B)$ *has parameters* $[[n, k, d]]$, *where*

$$n = 2\ell m, \quad k = 2 \cdot \dim\left(\mathsf{ker}(A) \cap \mathsf{ker}(B)\right), \quad \text{and} \quad d = \min\left\{|v| \colon v \in \mathsf{ker}(H^X) \backslash \mathsf{rs}(H^Z)\right\}.$$

The code offers equal distance for X*-type and* Z*-type errors.*

Proof. It is known [14, 15] that

$$k = n - \mathsf{rk}(H^X) - \mathsf{rk}(H^Z).$$

We claim that $\mathsf{rk}(H^X) = \mathsf{rk}(H^Z)$. Indeed, define a self-inverse permutation matrix C_ℓ of size $\ell \times \ell$ such that the i-th column of C_ℓ has a single nonzero entry equal to one at the row $j = -i \pmod{\ell}$. Define C_m similarly and let $C = C_\ell \otimes C_m$. Since $C_\ell S_\ell C_\ell = S_\ell^T$ and $C_m S_m C_m = S_m^T$, one gets

$$A^T = CAC \quad \text{and} \quad B^T = CBC. \tag{5}$$

Therefore one can write

$$H^Z = [B^T|A^T] = [CBC|CAC] = C[A|B] \begin{bmatrix} 0 & C \\ C & 0 \end{bmatrix} = CH^X \begin{bmatrix} 0 & C \\ C & 0 \end{bmatrix}.$$

Thus H^Z is obtained from H^X by multiplying on the left and on the right by invertible matrices. This implies $\mathsf{rk}(H^X) = \mathsf{rk}(H^Z)$. Therefore

$$\begin{aligned} k &= n - 2{\cdot}\mathsf{rk}(H^Z) = n - 2\Big(\frac{n}{2} - \dim\left(\mathsf{ker}((H^Z)^T)\right)\Big) = n - 2\Big(\frac{n}{2} - \dim\left(\mathsf{ker}(A) \cap \mathsf{ker}(B)\right)\Big) \\ &= 2 \cdot \dim\left(\mathsf{ker}(A) \cap \mathsf{ker}(B)\right). \end{aligned}$$

Here we noted that H^Z has size $(n/2) \times n$ and $\mathsf{ker}((H^Z)^T)) = \mathsf{ker}(A) \cap \mathsf{ker}(B)$ since $H^Z = [B^T|A^T]$.

It is known [14, 15] that a CSS code with check matrices H^X and H^Z has distance $d = \min(d^X, d^Z)$, where d^X and d^Z are the code distances for X-type and Z-type errors defined as

$$d^X = \min\big\{|v|\colon v \in \mathsf{ker}(H^Z)\backslash\mathsf{rs}(H^X)\big\} \quad \text{and} \quad d^Z = \min\big\{|v|\colon v \in \mathsf{ker}(H^X)\backslash\mathsf{rs}(H^Z)\big\}.$$

We claim that $d^Z \le d^X$. Indeed, let $X(f) = \prod_{j=1}^n X_j^{f_j}$ be a minimum weight logical X-type Pauli operator such that $|f| = d^X$. Then $H^Z f = 0$ and $f \notin \mathsf{rs}(H^X)$. Thus there exists a logical Z-type operator $Z(g) = \prod_{j=1}^n Z_j^{g_j}$ anti-commuting with $X(f)$. In other words, $H^X g = 0$ and $f^T g = 1$. Here, f and g are length-n binary vectors. Write $f = (\alpha, \beta)$ and $g = (\gamma, \delta)$, where $\alpha, \beta, \gamma, \delta$ are length-$(n/2)$ vectors. Conditions $H^Z f = 0$ and $H^X g = 0$ are equivalent to

$$B^T \alpha = A^T \beta \quad \text{and} \quad A\gamma = B\delta. \tag{6}$$

Here and below all arithmetics is modulo two. Define length-n vectors

$$e = (C\delta, C\gamma) \quad \text{and} \quad h = (C\beta, C\alpha). \tag{7}$$

From Eqs. (5,6) one gets

$$H^X h = [A|B] \begin{bmatrix} C\beta \\ C\alpha \end{bmatrix} = AC\beta + BC\alpha = C(A^T\beta + B^T\alpha) = 0.$$

Likewise,

$$H^Z e = [B^T|A^T] \begin{bmatrix} C\delta \\ C\gamma \end{bmatrix} = B^T C\delta + A^T C\gamma = C(B\delta + A\gamma) = 0.$$

Furthermore,

$$h^T e = \beta^T CC\delta + \alpha^T CC\gamma = \beta^T\delta + \alpha^T\gamma = f^T g = 1.$$

Thus $X(e)$ and $Z(h)$ are non-identity logical operators. It follows that $d^Z \le |h|$. We get

$$d^Z \le |h| = |C\beta| + |C\alpha| = |\beta| + |\alpha| = |f| = d^X.$$

Thus $d^Z \le d^X$. Similar argument shows that $d^X \le d^Z$, that is, $d^X = d^Z$. □

We note that the equality $d^X = d^Z$ can also be established using the machinery of Ref. [16] by viewing $\mathsf{QC}(A, B)$ as a Lifted Product code.

$[[n,k,d]]$	c_0	c_1	c_2
$[[72,12,6]]$	11.09	365.6	-16088
$[[90,8,10]]$	15.08	524.8	-12670
$[[108,8,10]]$	13.91	895	-46137
$[[144,12,12]]$	18.04	1337	-96007
$[[288,12,18]]$	32.04	3522	-294482

Table 3: Parameters c_0, c_1, c_2 in the fitting formula $p_L(p) = p^{d_{\mathsf{circ}}/2} e^{c_0+c_1p+c_2p^2}$ for BB LDPC codes shown in Table 1 in the main text.

4 Numerical simulation details

Data reported in Figure 3 A) in the main text was generated using BP-OSD software developed by Roffe et al. [5, 17]. The decoder was extended to the circuit-based noise model as described in Section 2. The simulations were performed using MIN-SUM belief propagation with the limit of $10,000$ iterations and combination sweep version of OSD, as described in [5]. All data points except for those with the smallest error rate accumulated at least 100 logical errors to estimate the logical error rate p_L with the error bars $\approx p_L/10$. The fitting formula $p_L(p) = p^{d_{\mathsf{circ}}/2} e^{c_0+c_1p+c_2p^2}$ with fitting parameters c_0, c_1, c_2 was proposed in [18] in the context of surface code simulations. We observed that the same fitting formula works well for BB LDPC codes. The fitting parameters c_i of the considered codes are provided in Table 3. We note that the logical error rate achieved by the combination of a distance-preserving SM circuit and an optimal decoder is expected to follow an exponential decay $p_L(p) = \exp[-d \cdot f(p)]$, where $f(p)$ is an unknown function such that $f(p) > 0$ in the sub-threshold regime. The function $f(p)$ must have a logarithmic singularity $f(p) \approx -(1/2)\log p$ for small p since one expects degree-$(d/2)$ error suppression for a distance-d code. The fitting formula for $p_L(p)$ approximates the remaining non-singular terms in $f(p)$ by a low-degree polynomial in p. Coefficients of the polynomial are considered as fitting parameters. Since our SM circuit is not distance-preserving, the code distance d in the fitting formula of Ref. [18] is replaced by the circuit-level distance d_{circ}.

Surface code data reported in Figure 3 B) in the main text was generated using software developed by one of the authors and Alexander Vargo in [18]. The simulation was performed for the rotated surface code with parameters $[[d^2, 1, d]]$, where $d \in \{9, 11, 13, 15\}$, and the standard SM circuit [3]. Let $P_{L,1}$ be the logical error probability for the surface code encoding one logical qubit and SM circuit with $N_c = d$ syndrome cycles. Encoding $k = 12$ logical qubits into 12 separate patches of the surface code results in a logical error probability

$$P_{L,12} = 1 - (1 - P_{L,1})^{12}.$$

Figure 3 B) in the main text shows the logical error rate p_L defined as the logical error probability per syndrome cycle,

$$p_L = 1 - (1 - P_{L,12})^{1/N_c} = 1 - (1 - P_{L,1})^{12/d}.$$

5 Logical memory capabilities

In this section we give evidence that BB LDPC codes have the required features for an effective quantum memory or storage unit. Although there are few ways of performing computations on stored qubits, there are fault tolerant operations for initialization and measurement of individual qubits, and most importantly transfer of data into and out of the code via quantum teleportation. These capabilities are based on a combination of two different techniques. First, we follow [19] to derive fault tolerant unitary operations that require only the connectivity already necessary to perform syndrome measurements. Second, we give low-overhead extensions of the Tanner graph based on work by [20] which enable measurement of a single logical operator while preserving the thickness-2 implementability criterion. Together, these capabilities allow us to address any logical qubit.

A conceptual representation of the logical operators is shown in Figure 1. We first derive logical Pauli operators for BB LDPC codes, and find that the logical qubits divide into an "unprimed" and a "primed" block with equal size

and symmetrical structure. We visualize the primed and unprimed block as two sheets featuring a 2D grid of logical operators. Some of these grid cells contain one of the $k/2$ logical qubits per sheet.

Next, we show that there exists a set of fault tolerant depth-four circuits that implement a small family of commuting logical CNOT circuits. These gates are based on automorphisms of the code: permutations of the data qubits that commute with the stabilizer. Based on their group structure we can think of the automorphism gates as translations of a 2D grid of operators within each of the primed and unprimed blocks. Furthermore, we follow [19] to derive a fault tolerant operation based on a ZX-duality that also allows us to swap the two blocks while also applying Hadamard gates to all qubits.

Finally, we show how to leverage techniques from [20] to extend the Tanner graph to a larger Tanner graph allowing fault-tolerant measurement of one logical X and one logical Z operator. Various subgraphs of this extended Tanner graph contain either this logical X or Z operator as a stabilizer. This construction acts as a "probe" that gives us access to one of the logical qubits.

Measurements of both logical X and Z operators on any qubit can be realized by conjugating this measurement by gates based on automorphisms and the ZX-duality. We can think of this as shifting any desired qubit to be the target of the probe using translation and exchange of the two blocks.

Logical X and Z measurement of any logical qubit also enables transfer of data into and out of the code using a teleportation circuit. This teleportation can be realized through a product measurement of the logical Pauli in the BB code, and a logical Pauli in another quantum error correction code. While the Tanner graph of the BB code demands thickness-2, we show how the ancilla system corresponding to the logical X measurement can be implemented in an "effectively planar" Tanner graph. This makes it possible two connect this ancilla system to another quantum error correction code, like a surface code, within a thickness-2 implementation. This capability indicates the suitability of BB LDPC codes as a fault tolerant quantum memory.

On the other hand, this construction incurs rather significant resource overhead that undercuts the compactness of the error correction codes introduced in this paper. For example, to equip the $[[144, 12, 12]]$ code with ancilla

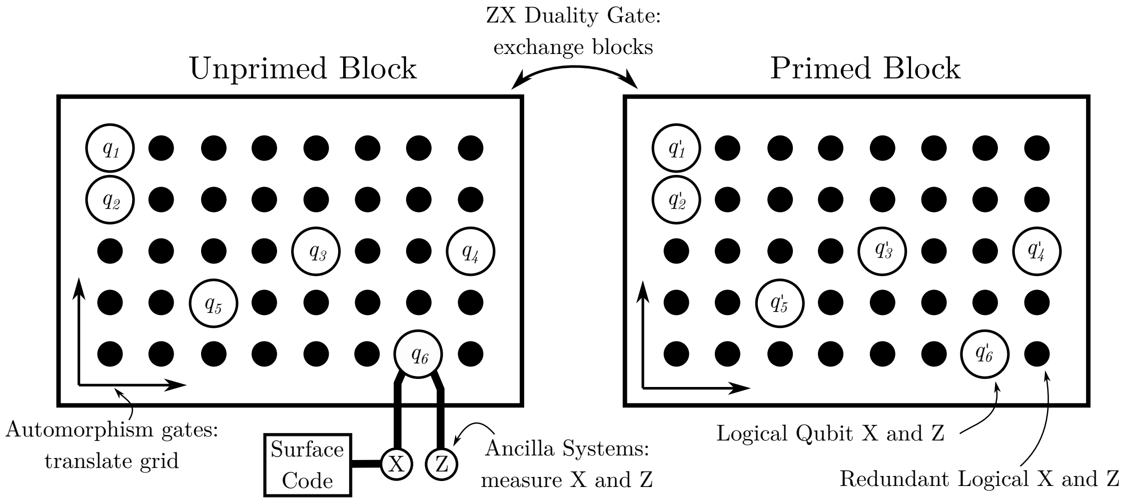

Figure 1: Conceptual diagram depicting the manner by which logical operators can be loaded into and out of a BB LDPC code. In Subsection 5.1 we derive that there are two blocks of logical Pauli operators corresponding to a 2d grid. Some subset of these grid elements can be chosen as logical qubits (large dots) and the other elements correspond to various Pauli products (small dots). In Subsection 5.2 we show that there are fault tolerant logical gates based on automorphisms that translate the grid of operators within each block, and in Subsection 5.3 we give a fault tolerant gate based on a ZX-duality [19] that swaps the two blocks. Finally, in Subsection 5.4 we show that there exists an ancilla system based on [20] that can measure one logical X operator. We can think of this system as a probe that can access one logical qubit. Together, these operations allow external access of every logical qubit and many of their products.

systems capable of measuring X and Z, we require $2 \times 30 \times (2d-1) = 1380$ additional qubits on top of the original 288. However, the argument for fault-tolerance from [20] is designed to be very broadly applicable, and hence may demand excessively many resources for any particular error correction code. We consider it very likely that the size of these systems can be significantly reduced. We leave resource optimization of this scheme for future work.

5.1 Logical Pauli Operators

In this section we derive that the logical Pauli matrices of BB LDPC codes split into a "primed" and an "unprimed" block with $|\mathcal{M}| = \ell m$ many X operators and Z operators each. Operators in the primed block commute with operators in the unprimed block, and the two blocks have identical commutation structure.

We begin by introducing some new notation for Pauli matrices acting on the data qubits. We denote with $\mathbb{F}_2^{\mathcal{M}}$ the set of polynomials over $\mathbb{F}_2$ with monomials from $\mathcal{M}$. This is equivalent to the quotient ring obtained from $\mathbb{F}_2[x,y]$ by identifying $x^\ell = y^m = 1$. With $x = S_m \otimes I$ and $y = I \otimes S_\ell$, the elements of $\mathbb{F}_2^{\mathcal{M}}$ have natural matrix representations, and can also be interpreted as sets since the coefficient on any particular monomial is either 0 or 1.

For $P, Q \in \mathbb{F}_2^{\mathcal{M}}$, we can consider the set of qubits $q(L,\alpha)$ for $\alpha \in P$ and $q(R,\beta)$ for $\beta \in Q$. We write $X(P,Q)$ to denote a Pauli matrix acting as X on this collection of qubits, and identity elsewhere. Similarly, $Z(P,Q)$ denotes Z acting on $q(L,\alpha)$ for $\alpha \in P$ and $q(R,\beta)$ for $\beta \in Q$. For example, we can recall that $q(L,\beta)$ is connected to $q(X,\alpha)$ whenever $\beta \in A\alpha$, and see that the stabilizer corresponding to $q(X,\alpha)$ becomes $X(\alpha A, \alpha B)$. Similarly, the stabilizer corresponding to $q(Z,\alpha)$ can be written as $Z(\alpha B^T, \alpha A^T)$. There is also the following useful fact:

Lemma 2. *$X(P,Q)$ anticommutes with $Z(\bar{P},\bar{Q})$ if and only if $1 \in P\bar{P}^T + Q\bar{Q}^T$.*

Proof. Write

$$P = \sum_{\alpha \in \mathcal{M}} p_\alpha \alpha, \quad \bar{P} = \sum_{\alpha \in \mathcal{M}} \bar{p}_\alpha \alpha, \quad Q = \sum_{\alpha \in \mathcal{M}} q_\alpha \alpha, \quad \bar{Q} = \sum_{\alpha \in \mathcal{M}} \bar{q}_\alpha \alpha,$$

where $p_\alpha, \bar{p}_\alpha, q_\alpha, \bar{q}_\alpha \in \mathbb{F}_2$ are coefficients. Pauli operators $X(P,Q)$ and $Z(\bar{P},\bar{Q})$ overlap on a qubit $q(L,\alpha)$ iff $p_\alpha \bar{p}_\alpha = 1$ and overlap on a qubit $q(R,\alpha)$ iff $q_\alpha \bar{q}_\alpha = 1$. Thus $X(P,Q)$ and $Z(\bar{P},\bar{Q})$ anti-commute iff $\sum_{\alpha \in \mathcal{M}} p_\alpha \bar{p}_\alpha + q_\alpha \bar{q}_\alpha$ is odd. We have

$$P\bar{P}^T = \sum_{\alpha \in \mathcal{M}} p_\alpha \bar{p}_\alpha 1 + \dots \quad \text{and} \quad Q\bar{Q}^T = \sum_{\alpha \in \mathcal{M}} q_\alpha \bar{q}_\alpha 1 + \dots$$

where dots represent all monomials different from 1. By linearity,

$$P\bar{P}^T + Q\bar{Q}^T = \sum_{\alpha \in \mathcal{M}} (p_\alpha \bar{p}_\alpha + q_\alpha \bar{q}_\alpha) 1 + \dots.$$

Thus $X(P,Q)$ and $Z(\bar{P},\bar{Q})$ anti-commute iff $P\bar{P}^T + Q\bar{Q}^T$ contains the monomial 1. □

Without loss of generality, we can express logical Pauli matrices as either $X(P,Q)$ or $Z(Q^T,P^T)$ via a choice of $P,Q \in \mathbb{F}_2^{\mathcal{M}}$. The operator $X(P,Q)$ commutes with the stabilizer $Z(\alpha B^T, \alpha A^T)$ whenever $1 \notin P(\alpha B^T)^T + Q(\alpha A^T)^T = \alpha^T(PB + QA)$. This is equivalent to $\alpha \notin PB + QA$. Since we must have $\alpha \notin PB + QA$ for all α, we see that $X(P,Q)$ commutes with the stabilizer whenever $PB + QA$ vanishes. Similarly we can derive that $Z(Q^T,P^T)$ commutes with the stabilizer when $PB + QA = 0$.

We aim to construct a family of solutions to $PB + QA = 0$ which give rise to a basis of logical qubits defined by a set of operators $\{\bar{X}_1, \bar{X}_2, ..., \bar{Z}_1, \bar{Z}_2, ...\}$ with the correct commutation relations. To do so, let us make some observations about Pauli operators defined via solutions to $PB + QA = 0$. First, if P,Q are a solution, then so are $\alpha P, \alpha Q$ for any $\alpha \in \mathcal{M}$, so each P,Q immediately gives rise to a family of $|\mathcal{M}| = lm$ logical operators for both X and Z. Second, consider using the same P,Q to define both $X(\alpha P, \alpha Q)$ and $Z(\beta Q^T, \beta P^T)$. Then these operators always commute because $\beta \alpha^T \in PQ + QP = 0$ never holds. So we require at least two solutions to $PB + QA = 0$ to define a set of operators with nontrivial commutation relations.

For reasons described later in Subsection 5.4, we would like a logical X operator with no support on $q(R)$. To this end, we select $f,g,h \in \mathbb{F}_2^{\mathcal{M}}$ that satisfy $Bf = 0$ and $gB + hA = 0$, yielding two solutions to the equation $PB + QA = 0$ with $P,Q = f,0$ and $P,Q = g,h$. These yield the following family of logical operators for all $\alpha \in \mathcal{M}$:

$$\begin{aligned} \bar{X}_\alpha &:= X(\alpha f, 0) & \bar{Z}_\alpha &:= Z(\alpha h^T, \alpha g^T) \\ \bar{X}'_\alpha &:= X(\alpha g, \alpha h) & \bar{Z}'_\alpha &:= Z(0, \alpha f^T) \end{aligned} \tag{8}$$

Polynomials for Logical Pauli Matrices in the $[[144, 12, 12]]$ Code

f

y \ x	0	1	2	3	4	5	6	7	8	9	10	11
5												
4												
3		●				●		●				●
2												
1												
0	●	●	●	●			●	●	●	●		

g

y \ x	0	1	2	3	4	5	6	7	8	9	10	11
5												
4	●											
3			●									
2	●	●										
1			●									
0		●										

h

y \ x	0	1	2	3	4	5	6	7	8	9	10	11
5												
4												
3	●	●										
2	●											
1	●	●										
0	●											

Table 4: Choices of polynomials f, g, h such that $\bar{X}_\alpha := X(\alpha f, 0)$ and $\bar{Z}_\alpha = Z(\alpha h^T, \alpha g^T)$ as defined in Eq. (8) are minimum-weight logical Pauli operators. The dots represent the monomials of the form $x^i y^j$ with coefficient 1. If we let $\{n_i\} = \{1, y, x^2y, x^2y^5, x^3y^2, x^4\}$ and $\{m_i\} = \{y, y^5, xy, 1, x^4, x^5y^2\}$, then $\bar{X}_{n_i}, \bar{Z}_{m_j}$ anticommute exactly when $i = j$. When used to construct an ancilla system as in Subsection 5.4, these polynomials give a system with 60 qubits per layer.

For all α, β, we see that $\bar{X}_\alpha, \bar{Z}'_\beta$ always commute because $f0^T + 0f^T = 0$, and $\bar{X}'_\beta, \bar{Z}_\alpha$ always commute because $gh + hg = 0$. Furthermore, $\bar{X}_\alpha, \bar{Z}_\beta$ and $\bar{X}'_\alpha, \bar{Z}'_\beta$ form anticommuting pairs when $\alpha^T\beta \in fh$. We see that we have constructed two independent blocks of logical operators with symmetrical structure. It follows that each of these blocks must contain a set of operators that define $k/2$ qubits. We name these the "unprimed" and "primed" logical blocks with $\bar{X}_\alpha, \bar{Z}_\beta$ and $\bar{X}'_\alpha, \bar{Z}'_\beta$ respectively.

Not all choices of f, g, h span all k logical qubits, but valid choices are readily enumerated in software. Solutions to $Bf = 0$ and $gB + hA = 0$ correspond to null spaces of B and $\begin{bmatrix} B \\ A \end{bmatrix}$ respectively, which can be constructed by Gaussian elimination. Gaussian elimination can also be used to check if the operators $\bar{X}_\alpha, \bar{Z}_\alpha, \bar{X}'_\alpha, \bar{Z}'_\alpha$ together span k qubits up to the stabilizer. We find all codes in Table 2 in the main text admit several such choices of f, g, h. In Table 4 we show a particularly favorable choice of f, g, h for the $[[144, 12, 12]]$ code where the resulting logical operators have minimum weight.

To identify logical qubits we can enumerate choices of monomials $\{n_1, n_2, \ldots, n_{k/2}\}$ and $\{m_1, m_2, \ldots, m_{k/2}\}$ such that $n_i^T m_j \in fh$ exactly when $i = j$. That way, $\bar{X}_{n_i}, \bar{Z}_{m_i}$ as well as $\bar{X}'_{n_i}, \bar{Z}'_{m_i}$ for $i = 1 \ldots k/2$ form a set of k logical qubits: $\bar{X}_{n_i}$ anticommutes with $\bar{Z}_{m_j}$ exactly when $i = j$. A brute force search readily finds choices of $\{n_i\}, \{m_i\}$.

5.2 Logical Gates based on Automorphisms

An automorphism of an error correction code is a permutation of the physical qubits that is equivalent to a permutation of the checks (more generally, an automorphism can map a check operator to a product of check operators). We focus on permutations that are implementable using fault tolerant circuits within the connectivity already required for syndrome measurements.

The existing connectivity admits some natural fault tolerant circuits implementing a particular family of permutations on the data qubits. BB LDPC codes feature two data registers $q(L), q(R)$ and two check registers $q(X), q(Z)$. We consider circuits that transfer the qubits from the data registers to the ancilla registers, and back again on a different path. The adjacency matrices describing the connectivity between the data and the ancilla registers are given by A and B, which are the sum of three monomials A_1, A_2, A_3 and B_1, B_2, B_3 in $\mathcal{M}$. Each monomial is a permutation and thus describes a vertex-disjoint set of edges between the data and ancilla block. Hence, all swaps along these edges can be parallelized. In a single circuit we can either swap along the edges defined by A which are $q(L) \leftrightarrow q(X)$ and $q(R) \leftrightarrow q(Z)$, or along edges defined by B which are $q(L) \leftrightarrow q(Z)$ and $q(R) \leftrightarrow q(X)$. See also Figure 5 A) in the main text.

The monomial defining the particular set of edges in each of these sets of swaps can be chosen independently for each stage of the permutation (data $\rightarrow$ ancilla or ancilla $\rightarrow$ data), and on each side of the Tanner graph. For example, we can

'A' type automorphism based on any A_j, A_k

for $\alpha \in \mathcal{M}$ **do**
  InitZ $q(X, \alpha)$
  CNOT $q(L, A_j\alpha)$ $q(X, \alpha)$
  CNOT $q(X, \alpha)$ $q(L, A_j\alpha)$
  InitZ $q(L, A_k^T\alpha)$
  CNOT $q(X, \alpha)$ $q(L, A_k\alpha)$
  CNOT $q(L, A_k\alpha)$ $q(X, \alpha)$
end for

for $\alpha \in \mathcal{M}$ **do**
  InitZ $q(Z, \alpha)$
  CNOT $q(R, \alpha)$ $q(Z, A_j\alpha)$
  CNOT $q(Z, A_j\alpha)$ $q(R, \alpha)$
  InitZ $q(R, A_k\alpha)$
  CNOT $q(Z, A_k\alpha)$ $q(R, \alpha)$
  CNOT $q(R, \alpha)$ $q(Z, A_k\alpha)$
end for

'B' type automorphism based on any B_j, B_k

for $\alpha \in \mathcal{M}$ **do**
  InitZ $q(X, \alpha)$
  CNOT $q(R, B_j\alpha)$ $q(X, \alpha)$
  CNOT $q(X, \alpha)$ $q(R, B_j\alpha)$
  InitZ $q(R, B_k^T\alpha)$
  CNOT $q(X, \alpha)$ $q(R, B_k\alpha)$
  CNOT $q(R, B_k\alpha)$ $q(X, \alpha)$
end for

for $\alpha \in \mathcal{M}$ **do**
  InitZ $q(Z, \alpha)$
  CNOT $q(L, \alpha)$ $q(Z, B_j\alpha)$
  CNOT $q(Z, B_j\alpha)$ $q(L, \alpha)$
  InitZ $q(L, B_k\alpha)$
  CNOT $q(Z, B_k\alpha)$ $q(L, \alpha)$
  CNOT $q(L, \alpha)$ $q(Z, B_k\alpha)$
end for

Table 5: Circuits implementing automorphisms of a BB LDPC code within the connectivity already present for syndrome checks. These circuits are fault tolerant and have CNOT depth four. If $s = A_jA_k^T$ or $s = B_jB_k^T$, then the logical gate implemented by these automorphisms performs the transformation $\bar{X}_\alpha, \bar{Z}_\alpha, \bar{X}'_\alpha, \bar{Z}'_\alpha \to \bar{X}_{s\alpha}, \bar{Z}_{s\alpha}, \bar{X}'_{s\alpha}, \bar{Z}'_{s\alpha}$.

select any $A_j, A_k, A_{j'}, A_{k'}$ and move $q(L) \to_{A_j^T} q(X) \to_{A_k} q(L)$ and simultaneously move $q(R) \to_{A_{k'}} q(Z) \to_{A_{j'}^T} q(R)$. However, we will see later that it is necessary to select $A_j = A_{j'}$ and $A_k = A_{k'}$. Furthermore, these swaps admit a standard optimization: if we initialize the check registers $q(X), q(Z)$ to the $|0\rangle$ state, then circuits implementing these permutations have CNOT depth four. If we also reset qubits to the $|0\rangle$ state in between the swaps wherever possible, we obtain circuits whose errors cannot propagate between physical qubits, and are hence fault tolerant. See Table 5.

We now verify that the permutations implemented by the circuits described above are indeed automorphisms. After having applied an 'A' type permutation based on A_j, A_k, the qubits are permuted by $q(L, \alpha) \leftrightarrow q(L, A_k^T A_j\alpha)$ and $q(R, \alpha) \leftrightarrow q(R, A_k^T A_j\alpha)$. We see that this transforms a Pauli matrix by $X(P, Q) \to X(A_jA_k^T P, A_jA_k^T Q)$. Consequently, the stabilizers are transformed as $X(\alpha A, \alpha B) \to X(\alpha A_jA_k^T A, \alpha A_jA_k^T B)$, which is the same as permuting the X checks by $\alpha \to \alpha A_jA_k^T$. The Z stabilizers are also permuted by $\alpha \to \alpha A_jA_k^T$, so the described circuit indeed implements an automorphism. Notice also that this only works because the $q(L)$ and $q(R)$ blocks were transformed by the same $A_jA_k^T$. The 'B' type permutations can be verified to be automorphisms in the same manner, permuting the checks by some $B_jB_k^T$.

These automorphisms allow us to fault tolerantly implement a subgroup of the Clifford gates. As we saw in Lemma 3 in the main text, shifts of the form $A_jA_k^T$ or $B_jB_k^T$ generate the entire group $\mathcal{M}$ whenever the Tanner graph is connected. Therefore, by leveraging these permutations as generators, we can perform all translations of the tori containing $q(L), q(R)$ using fault tolerant circuits of varying depth. An automorphism defined by an element $s \in \mathcal{M}$ transforms $\bar{X}_{n_i} \to \bar{X}_{sn_i}$, $\bar{Z}_{m_i} \to \bar{Z}_{sm_i}$ and similarly for the primed logical Pauli matrices. This capability is critical for addressing all logical qubits.

We can also comment on the nature of these operations as logical gates, although they are less useful in this sense. There is one such operation per element in $\mathcal{M}$, and since $\mathcal{M}$ is Abelian the subgroup of Clifford gates implemented by these automorphisms must be Abelian as well. A transformation of this form cannot act like the logical identity so all of these gates (except $s = 1$) are nontrivial. Since automorphism operations take $\bar{X}$ to $\bar{X}$ and $\bar{Z}$ to $\bar{Z}$, and they must hence be logical CNOT circuits up to a logical Pauli correction. While it is not clear how to use these CNOT circuits to facilitate useful computations, they may make for interesting test cases in an implementation.

5.3 Accessing the Primed Block via a ZX-duality

A ZX-duality is a permutation of the logical qubits that commutes with the stabilizer, except that it turns X checks into Z checks and Z checks into X checks. A physical circuit implementing this permutation and then applying Hadamard to all data qubits always acts as a logical gate [19]. In this section we focus on the implementation of a particular ZX-duality with applications for readout. We leave discovery and implementation of other ZX-dualities for future work. In particular, we derive a general method for constructing fault tolerant circuits for implementing a particular ZX-duality that is present in all BB LDPC codes. While the circuits from this construction are generally quite expensive, they may be amenable to further optimization and can be used sparingly in practice.

Consider a permutation of data qubits that swaps $q(L, \alpha)$ with $q(R, \alpha^T)$ for all $\alpha \in \mathcal{M}$. A check qubit $q(X, \beta)$ which previously implemented the stabilizer $X(\beta A, \beta B)$ now is connected to the qubits $q(L, (\beta B)^T)$ and $q(R, (\beta A)^T)$ instead, corresponding to the check $Z(\beta^T B^T, \beta^T A^T)$. We see that this permutation switches the stabilizer implemented by $q(X, \beta)$ with the stabilizer implemented by $q(Z, \beta^T)$, so this permutation is indeed a ZX-duality.

We can also see that implementing this permutation and applying Hadamard to all qubits takes logical Pauli matrices to logical Pauli matrices. In particular, the operation swaps $\bar{X}_\alpha = X(\alpha f, 0)$ with $\bar{Z}'_{\alpha^T} = Z(0, \alpha^T f^T)$, as well as $\bar{Z}_\alpha = Z(\alpha h^T, \alpha g^T)$ with $\bar{X}'_{\alpha^T} := X(\alpha^T g, \alpha^T h)$. This operation swaps the primed and unprimed logical blocks, transposes the grid of operators, and applies logical Hadamard to all qubits. Since we can measure logical X for all qubits in the unprimed block using the ancilla system described in Subsection 5.4, we can use this operation to measure logical Z for qubits in the primed block.

For the rest of this section we describe a fault tolerant method for implementing this operation. We begin with exchanging $q(L)$ and $q(R)$: since these blocks are connected by pairs of edges in $q(X)$ and $q(Z)$, for any $A_i \in A$ and $B_j \in B$ there exists a loop connecting the qubits $q(L, \alpha) \to q(X, A_i^T \alpha) \to q(R, B_j A_i^T \alpha) \to q(Z, B_j \alpha) \to q(L, \alpha)$. A circuit identical in shape to those in Table 5 hence performs a fault tolerant exchange of $q(L, \alpha)$ and $q(R, B_j A_i^T \alpha)$ for all α. The additional shift of $B_j A_i^T$ can be removed via an additional automorphism gate. It remains to exchange $q(L, \alpha) \leftrightarrow q(L, \alpha^T)$, as well as $q(R, \alpha) \leftrightarrow q(R, \alpha^T)$ for all α, which is significantly more complicated. We focus on $q(L, \alpha) \leftrightarrow q(L, \alpha^T)$ in our discussion but it will be clear the exact same transformations are implementable on $q(R)$ in parallel with those on $q(L)$.

Fault tolerant implementation of the permutation $q(L, \alpha) \leftrightarrow q(L, \alpha^T)$ can be achieved using a more sophisticated version of the fault tolerant circuits in Table 5 used for implementing automorphisms. These circuits relied on the existence of a connected loop of alternating check and data qubits, enabling a short depth fault tolerant circuit implementing a cyclic permutation of the data qubits therein. The same connectivity can be leveraged to implement a fault tolerant nearest neighbor swap of two data qubits connected by a check qubit. The fault tolerance of these circuits relies on the same principle: while a swap gate acting on two qubits containing data is not fault tolerant, moving a data qubit onto a blank qubit is. Figure 2 A) shows a subgraph of the Tanner graph consisting of several connected qubits in the $q(L)$ and $q(X)$ block, and Figure 2 B) shows a sequence of operations where two data qubits can be exchanged without ever interacting directly. This gives us the following capability: whenever the circuits in Table 5 can implement the cyclic permutation $q(L, \alpha) \to q(L, s\alpha)$ for all α, there also exists a circuit that can swap $q(L, \alpha)$ and $q(L, s\alpha)$ for a particular α. Matching circuits exist for $q(R)$, and can be implemented simultaneously.

To decompose $q(L, \alpha) \leftrightarrow q(L, \alpha^T)$ into a sequence of swaps, it will be helpful to consider the group structure of $\mathcal{M}$. Consider for example the [[90, 8, 10]] code with $x^{15} = y^3 = 1$. Following the classification of finite Abelian groups we see that $\mathcal{M} \cong \mathbb{Z}_3 \times \mathbb{Z}_5 \times \mathbb{Z}_3$. We can re-express elements of $\mathcal{M}$ using generators p, q, r with $p^3 = q^5 = r^3 = 1$ where $x = pq$ and $y = r$. Transforming α to α^T amounts to decomposing α as $\alpha = p^i q^j r^k$ and exchanging the qubit with $\alpha^T = p^{-i} q^{-j} r^{-k}$.

This exchange $p^i q^j r^k \leftrightarrow p^{-i} q^{-j} r^{-k}$ can be split into a sequence of swaps that are implementable with the method described above using Figure 2 A) and B). It suffices to be able to exchange for any $\alpha = q^j r^k$ the qubits $q(L, p^i \alpha) \leftrightarrow q(L, \alpha p^{-i})$, as well as for any $\alpha = p^i r^k$ the qubits $q(L, q^j \alpha) \leftrightarrow q(L, \alpha q^{-j})$, and finally for any $\alpha = p^i q^j$ the qubits $q(L, r^k \alpha) \leftrightarrow q(L, \alpha r^{-k})$. This, for any i, j, k, creates a sequence of qubits $q(L, p^i q^j r^k) \leftrightarrow q(L, p^{-i} q^j r^k) \leftrightarrow q(L, p^{-i} q^{-j} r^k) \leftrightarrow q(L, p^{-i} q^{-j} r^{-k})$ where swaps are possible along each nearest neighbor. This is sufficient for swapping the first and last qubit in the chain. The implementation of the individual generators like $q(L, \alpha q^i) \leftrightarrow q(L, \alpha q^{-i})$ swaps may also involve additional intermediate qubits, but this only lengthens the chain and does not prohibit implementation.

The resources required for swapping $q(L, p^i \alpha) \leftrightarrow q(L, \alpha p^{-i})$ where $p^3 = 1$ and similarly for other generators depends on the order of the generator p as well as the ratios $A_i A_j^T$ that can be formed using terms $A_i, A_j \in A$ or

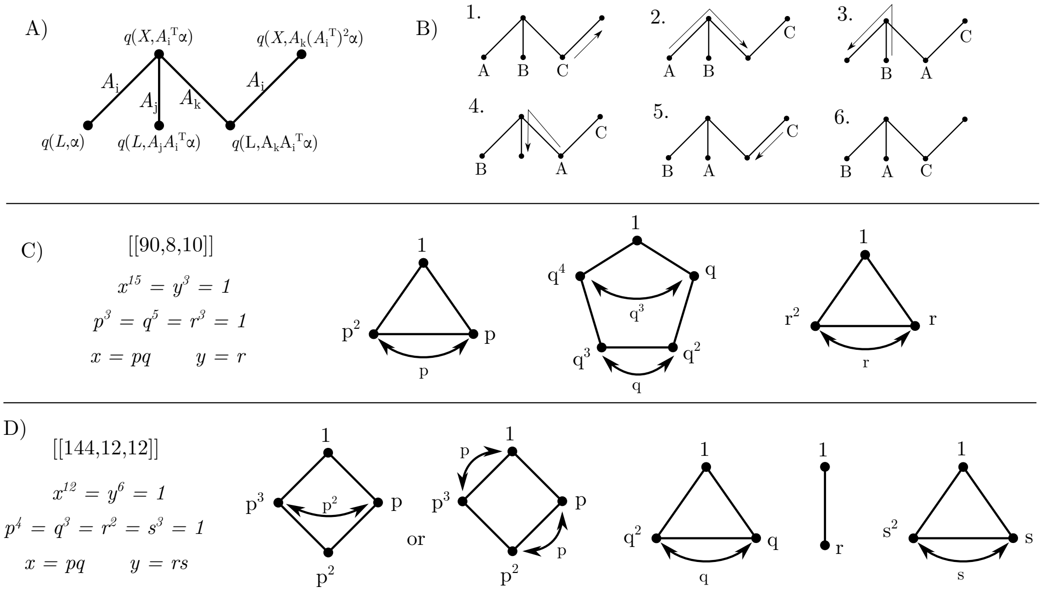

Figure 2: Diagrams for the description of the implementation of the ZX-duality permutation. A) A subgraph of the Tanner graph providing enough connectivity to fault tolerantly swap $q(L,\alpha)$ and $q(L,A_jA_i^T\alpha)$. B) A sequence of shifts of the data on the qubits in the $q(L)$ block that performs the desired exchange without interacting qubits directly. A naive implementation of this sequence has CNOT depth 12. C) D) Decomposition of the generators of $\mathcal{M}$ via the classification of finite Abelian groups for two different codes. Drawing the Cayley graph of the subgroup for each generator reveals the ratios defining pairs of qubits that must be exchanged to implement the permutation $q(L,\alpha) \leftrightarrow q(L,\alpha^T)$.

similar ratios from B. See Figure 2 C). Plotting the Cayley graph of the cyclic subgroup spanned by p immediately reveals that since p is order three, only a single ratio $B_iB_j^T = p$ is needed in order to swap any qubits marked p^1 and p^2, while leaving p^0 qubits in place. Indeed since $B = 1 + x^2 + x^7 = 1 + p^2q^2 + pq^2$ we can implement $p = (p^2q^2)(pq^2)^T$ in a single layer of transforms in Figure 2 B).

The exchange $q(L,\alpha q^i) \leftrightarrow q(L,\alpha q^{-i})$ with $q^5 = 1$ requires two such ratios q and q^2, the minimal depth expression of which demands the chaining together of two such transforms each. In other codes, like the [[144, 12, 12]] code, we encounter generators p, q, r, s of order $p^4 = q^3 = r^2 = s^3 = 1$. Elements of order two like r require no swaps at all, and elements of order four like p can be implemented either using the ratio p^2 or just p, as shown in Figure 2 D). Numerical searches can quickly compute the most efficient decompositions of the required swaps. We give the orders of the generators, the ratios defining the required swaps, and the number of transforms required to implement them in Table 6.

We emphasize that the swap $q(L,p^i\alpha) \leftrightarrow q(L,\alpha p^{-i})$ can be performed for all $\alpha = q^jr^k$ simultaneously in parallel. This stems from the structure of the exchange circuit in Figure 2 B). This circuit performs the swap $q(L,\alpha) \leftrightarrow q(L,A_jA_i^T\cdot\alpha)$ while using the qubit $q(L,A_kA_i^T\cdot\alpha)$ as scratch space. However, we can simultaneously want to swap $q(L,A_kA_i^T\cdot\alpha) \leftrightarrow q(L,A_jA_i^T\cdot A_kA_i^T\cdot\alpha)$ since the first step of the exchange circuit in Figure 2 B) is to move the data marked 'A' away from the qubit holding it, just as if it were a piece of data marked 'C' for a different exchange.

For clarity, we compute the total depth of the circuit for the [[144, 12, 12]] code without any further optimization. The ratios p, q, s can be implemented using two ratios each via $p = x^{-3}y\cdot y^{-2}y$, $q = y^{-3}x^2\cdot y^{-3}x^2$ and $s = y^{-2}y\cdot y^{-2}y$. This results in a chain $q(L,\alpha) \leftrightarrow q(L,\alpha') \leftrightarrow q(L,\alpha'')... \leftrightarrow q(L,\alpha^T)$ of length six (counting the number of $\leftrightarrow$s). We can swap the qubits at the ends of a chain of length n using $2n-1$ many nearest neighbor swaps. Each swap circuit of the form Figure 2 B) can be implemented in CNOT depth twelve, resulting in CNOT depth $(2\cdot 6 - 1)\cdot 12 = 132$ to implement $q(L,\alpha) \leftrightarrow q(L,\alpha^T)$.

Despite its fault tolerance, the implementation of this logical operation is clearly significantly more expensive than that of the automorphisms. Since the intermediate permutations corresponding to each of the generators p, q, r are

Code	Base Order	Reduced Order	Required Ratios	Swap Chain Length
$[[72, 12, 6]]$	x^6, y^6	p^2, q^3, r^2, s^3	q, s	4
$[[90, 8, 10]]$	x^{15}, y^3	p^3, q^5, r^3	p, q, q^2, r	6
$[[108, 8, 10]]$	x^9, y^6	p^9, q^2, r^3	p, p^3, p^5, p^7, r	9
$[[144, 12, 12]]$	x^{12}, y^6	p^4, q^3, r^2, s^3	p, q, s	6
$[[288, 12, 18]]$	x^{12}, y^{12}	p^4, q^3, r^4, s^3	p, q, r, s	10
$[[360, 12, \leq 24]]$	x^{30}, y^6	p^2, q^3, r^5, s^2, t^3	q, ps, psr^2, psr^3, t	11

Table 6: Table deriving the steps in the circuit implementing the $q(L, \alpha) \leftrightarrow q(L, \alpha^T)$, permutation for the ZX-duality for several codes. The generators of $\mathcal{M}$ are decomposed into generators following the decomposition of finite Abelian groups. Following Figure 2 B) and C) these generators demand a set of ratios of terms in A or B which define fault tolerantly implementable exchanges of qubits with corresponding labels. The result is a decomposition of $q(L, \alpha) \leftrightarrow q(L, \alpha^T)$ into a chain $q(L, \alpha) \leftrightarrow q(L, \alpha') \leftrightarrow q(L, \alpha'') \leftrightarrow ... \leftrightarrow q(L, \alpha^T)$ with length as shown (counting the number of arrows, $\leftrightarrow$). Note the special implementation of the ratios in the $[[360, 12, \leq 24]]$ code: the ratios r^2, r^3 are not implementable, but psr^2, psr^3 are. This is fine if we can also perform the ps ratio on its own to remove the additional transformation on some of the qubits.

not ZX dualities in general, it will not be possible in general to perform error correction during this long operation. However, the significant overhead of this operation may be worth such a large cost, since it grants us the capability of accessing the primed block of qubits, effectively doubling the storage capacity of the code. This operation can also be used significantly more sparingly than the automorphism gates, and may be amenable to additional optimization. Alternatively, additional connections and qubits beyond those necessary for the Tanner graph could be introduced to more directly implement the ZX-duality, though it is likely this will sacrifice the thickness-2 property.

5.4 Logical Measurements

In this section we describe how to leverage methods from [20] to implement fault-tolerant measurements of the operators $\bar{X}_1 = X(f, 0)$ and $\bar{Z}_1 = Z(h^T, g^T)$. As described above, this capability suffices to measure $\bar{X}$ and $\bar{Z}$ for all logical qubits. We can also use this technique to measure various Pauli product operators by measuring $\bar{X}_\alpha, \bar{Z}_\alpha, \bar{X}'_\alpha, \bar{Z}'_\alpha$ for α not corresponding to logical qubits.

The measurement is facilitated by an ancilla system that extends the Tanner graph of the original code. The code defined by this extended Tanner graph contains the logical operator of interest as a stabilizer, enabling its fault tolerant measurement. A sketch of the structure of this ancilla system is given in Figure 1 C) in the main text. For the logical operator $X(f, 0)$, we consider a subgraph of the Tanner graph consisting of $q(L, f)$ as well as $q(Z, \alpha)$ operators corresponding to checks with support on $q(L, f)$. Similarly for the logical operator $Z(h^T, g^T)$ we consider a subgraph consisting of $q(L, h^T), q(R, g^T)$ as well as $q(X, \alpha)$ for the relevant α. These subgraphs are copied several times and are connected together as shown in the figure: we call the resulting construction an ancilla system. With enough copies, the code defined by the extended Tanner graph has the same distance as the original code.

Furthermore, the ancilla system for measuring $X(f, 0)$ can be connected to another quantum error correction code, such as a surface code. This enables a joint $\bar{X}\bar{X}$ measurement between a surface code qubit and any qubit within the BB code. A subsequent measurement of $Z(h^T, g^T)$ and some additional Pauli corrections then achieves a quantum teleportation circuit.

The main challenge of implementing these ancilla systems, in addition to minimizing their size, is to show that the extended Tanner graph satisfies the thickness-2 constraint. If our goal is to leverage the $X(f, 0)$ ancilla system to measure a Pauli product measurement with a surface code qubit, then arguably a thickness-2 extension of the Tanner graph does not suffice since there is no obvious way of connecting it to the surface code qubit as in Figure 1 C) in the main text. To this end, we show how to make the subgraph corresponding to the $X(f, 0)$ ancilla system "effectively planar": while the graph has thickness-2, the planar graph in one plane consists entirely of connected components with two vertices. Given this property of the embedding of the $X(f, 0)$ ancilla system, a connection between this system and a surface code may be facilitated by a construction that is thickness-2 overall.

An effectively planar embedding of the ancilla system relies on the fact that the logical operator $X(f, 0)$ has no

support on the $q(R)$ block. An implementation of more general logical operators is possible, but would require a graph that renders many ancilla qubits inaccessible from the outside.

We briefly give a self-contained description for the construction of the ancilla system from [20], following their notation. Suppose we are interested in measuring a logical operator $\bar{X}$ that is supported on some set of qubits $V_{\bar{X}}$. Then, let $C_{\bar{X}}$ be the collection of Pauli-Z checks that have support on any of the $V_{\bar{X}}$. If we view these as sets of vertices in a Tanner graph, and let $E_{\bar{X}}$ contain the edges between $V_{\bar{X}}$ and $C_{\bar{X}}$, then $\mathcal{G}_{\bar{X}} := (V_{\bar{X}}, C_{\bar{X}}, E_{\bar{X}})$ forms a subgraph of the Tanner graph of the BB code.

The ancilla system is constructed out of copies of 'primal layers' isomorphic to $\mathcal{G}_{\bar{X}}$, and 'dual layers' isomorphic to $\mathcal{G}^T_{\bar{X}} := (V^T_{\bar{X}}, C^T_{\bar{X}}, E^T_{\bar{X}})$ defined as follows: each $v \in V_{\bar{X}}$ has a corresponding $v^T \in C^T_{\bar{X}}$, each $c \in C_{\bar{X}}$ has a corresponding $c^T \in V^T_{\bar{X}}$, and each edge $(v, c) \in E_{\bar{X}}$ has a corresponding $(v^T, c^T) \in E^T_{\bar{X}}$. For some parameter r, the final Tanner graph is that of the BB code, plus r additional copies of the dual graph labeled $\mathcal{G}^T_{\bar{X}}[j]$ for $1 \leq j \leq r$, and $r-1$ additional copies of the primal graph labeled $\mathcal{G}_{\bar{X}}[j]$ for $2 \leq j \leq r$. We regard the $\mathcal{G}_{\bar{X}}$ within the original code as $\mathcal{G}_{\bar{X}}[1]$. We also add additional connections between $\mathcal{G}_{\bar{X}}[j]$ and $\mathcal{G}^T_{\bar{X}}[j]$ for $j \leq r$, as well as $\mathcal{G}^T_{\bar{X}}[j]$ and $\mathcal{G}_{\bar{X}}[j+1]$ for $j < r$: specifically, we connect the associated pairs of v, v^T and c, c^T.

It is shown by [20] that the resulting Tanner graph defines an error correction code of distance d when $r = d$. We construct two such ancilla systems: one for $\bar{X} := X(f, 0)$ and one for $\bar{Z} := Z(h^T, g^T)$. Table 4 shows a choice of f, g, h for the $[[144, 12, 12]]$ code, defining $X(f, 0)$ and $Z(h^T, g^T)$ such that these operators are all minimum weight, and define $\mathcal{G}_{\bar{X}}$ and $\mathcal{G}_{\bar{Z}}$ with 30 qubits each. To achieve $d = 12$ we hence require $2 \times 30 \times (2d - 1) = 1380$ additional qubits. We suspect that significantly more efficient variations of this constructions are possible, but leave their development for future work.

The construction presented above is complicated by the fact that vertices in the Tanner graph take on alternating roles in each layer: in the primal layers the vertices v are physical qubits, whereas in the dual layers the v^T are checks. However, for the purposes of giving a thickness-2 decomposition we need not concern ourselves with this. If we do not distinguish between checks and physical qubits, then the primal layers $\mathcal{G}_{\mathcal{X}}$ and dual layers $\mathcal{G}^T_{\mathcal{X}}$ have isomorphic Tanner graphs. Hence, for the purposes of the following, we view all layers as identical.

We now show why a thickness-2 embedding of the $Z(h^T, g^T)$ ancilla system, and an effectively planar embedding of the $X(f, 0)$ ancilla system is possible. This argument is best understood in reference to Figure 3. We begin by understanding the thickness-2 decomposition of each layer of the ancilla systems, leveraging Figure 4 in the main text. In Figure 3 A), we can see that $\mathcal{G}_{\bar{Z}}$ for the $Z(h^T, g^T)$ system decomposes into 'hairy rings' in both the 'A' plane and the 'B' plane since it has no support on $q(Z)$. $\mathcal{G}_{\bar{X}}$ for the $X(f, 0)$ system is a collection of connected pairs in the 'A' plane and collection of rings in the 'B' plane, since it has no support on $q(X)$.

Since $\mathcal{G}_{\bar{X}}, \mathcal{G}_{\bar{Z}}$ are subgraphs of the BB code's Tanner graph, and its Tanner graph has thickness-2, and since $\mathcal{G}_{\bar{X}}, \mathcal{G}_{\bar{Z}}$ and $\mathcal{G}^T_{\bar{X}}, \mathcal{G}^T_{\bar{Z}}$ are isomorphic if we do not distinguish between qubits and checks, we see that each layer of the ancilla construction must be thickness-2 individually. The main challenge is to show that the connections between the layers can be facilitated without introducing any crossings.

Figure 3 B) shows how to connect several layers of the two ancilla systems to both the wheel graphs of the BB code, and also an ancillary surface code. We arrange the wheels of the BB code such that $q(X), q(L)$ are on the inside of the 'A' wheels, and that $q(X), q(R)$ are on the inside of the 'B' wheels. $\mathcal{G}_{\bar{Z}}$ and $\mathcal{G}^T_{\bar{Z}}$ for of the $Z(h^T, g^T)$ system can be repeatedly nested inside of the wheels of the BB code. The $q(L)$ qubits can be connected together on the 'A' plane, and the $q(R)$ and $q(X)$ qubits can be connected on the 'B' plane. As for $\mathcal{G}_{\bar{X}}$ and $\mathcal{G}^T_{\bar{X}}$ for the $X(f, 0)$ system, the rings in the 'B' plane can be wrapped around the wheels of the BB code which already allows connection of the required $q(L)$ and $q(Z)$ qubits. This leaves the pairs of connected qubits in the 'A' plane completely free of any connections between the layers, making them available to be connected to a surface code system.

We have considered just two ancilla systems here for measuring $X(f, 0)$ and $Z(h^T, g^T)$. However, using additional ancilla systems, especially if their size can be reduced, or equipping these two ancilla systems with additional connections to the $X(g, h)$ and $Z(0, f^T)$ logical operators are potential ways to eliminate the need for the error-prone ZX-duality from Subsection 5.3 and access all logical qubits. On the other hand, it is not clear that either approach would preserve the thickness-2 property.

References

[1] Maxime A. Tremblay, Nicolas Delfosse, and Michael E. Beverland. Constant-overhead quantum error correction with thin planar connectivity. *Physical Review Letters*, 129(5):050504, 2022.

[2] Scott Aaronson and Daniel Gottesman. Improved simulation of stabilizer circuits. *Physical Review A*, 70(5):052328, 2004.

[3] Austin G. Fowler, Ashley M. Stephens, and Peter Groszkowski. High-threshold universal quantum computation on the surface code. *Physical Review A*, 80(5):052312, 2009.

[4] Pavel Panteleev and Gleb Kalachev. Degenerate quantum LDPC codes with good finite length performance. *Quantum*, 5:585, 2021.

[5] Joschka Roffe, David R. White, Simon Burton, and Earl Campbell. Decoding across the quantum low-density parity-check code landscape. *Physical Review Research*, 2(4):043423, 2020.

[6] Austin G. Fowler, Adam C. Whiteside, Angus L. McInnes, and Alimohammad Rabbani. Topological code autotune. *Physical Review X*, 2(4):041003, 2012.

[7] Oscar Higgott and Craig Gidney. Sparse blossom: correcting a million errors per core second with minimum-weight matching. *arXiv preprint arXiv:2303.15933*, 2023.

[8] Nicolas Delfosse and Adam Paetznick. Spacetime codes of clifford circuits. *arXiv preprint arXiv:2304.05943*, 2023.

[9] Matt McEwen, Dave Bacon, and Craig Gidney. Relaxing hardware requirements for surface code circuits using time-dynamics. *Quantum*, 7:1172, 2023.

[10] Oscar Higgott, Thomas C. Bohdanowicz, Aleksander Kubica, Steven T. Flammia, and Earl T. Campbell. Improved decoding of circuit noise and fragile boundaries of tailored surface codes. *Physical Review X*, 13(3):031007, 2023.

[11] Gyorgy P. Geher, Ophelia Crawford, and Earl T. Campbell. Tangling schedules eases hardware connectivity requirements for quantum error correction. *arXiv preprint arXiv:2307.10147*, 2023.

[12] Elwyn Berlekamp, Robert McEliece, and Henk Van Tilborg. On the inherent intractability of certain coding problems (corresp.). *IEEE Transactions on Information Theory*, 24(3):384–386, 1978.

[13] Andrew J. Landahl, Jonas T. Anderson, and Patrick R. Rice. Fault-tolerant quantum computing with color codes. *arXiv preprint arXiv:1108.5738*, 2011.

[14] Andrew Steane. Multiple-particle interference and quantum error correction. *Proceedings of the Royal Society of London. Series A: Mathematical, Physical and Engineering Sciences*, 452(1954):2551–2577, 1996.

[15] A. Robert Calderbank and Peter W. Shor. Good quantum error-correcting codes exist. *Physical Review A*, 54(2):1098, 1996.

[16] Pavel Panteleev and Gleb Kalachev. Quantum LDPC codes with almost linear minimum distance. *IEEE Transactions on Information Theory*, 68(1):213–229, 2021.

[17] Joschka Roffe. LDPC: Python tools for low density parity check codes, 2022.

[18] Sergey Bravyi and Alexander Vargo. Simulation of rare events in quantum error correction. *Physical Review A*, 88(6):062308, 2013.

[19] Nikolas P. Breuckmann and Simon Burton. Fold-transversal Clifford gates for quantum codes. *arXiv preprint arXiv:2202.06647*, 2022.

[20] Lawrence Z. Cohen, Isaac H. Kim, Stephen D. Bartlett, and Benjamin J. Brown. Low-overhead fault-tolerant quantum computing using long-range connectivity. *Science Advances*, 8(20), 2022.

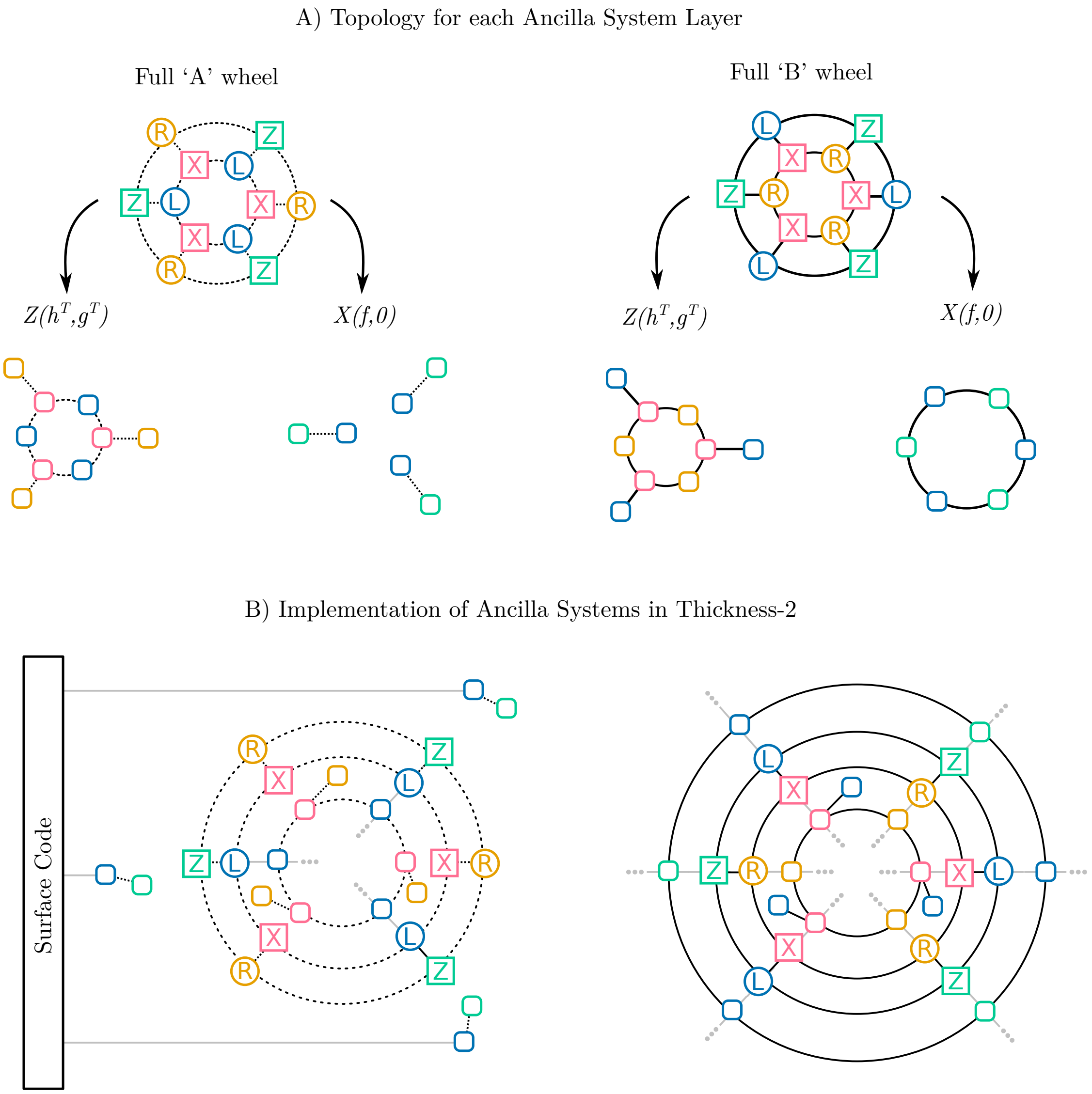

Figure 3: Illustration of the thickness-2 property of the Tanner graph of BB LDPC codes. A) Planar embedding of each layer of the ancilla system from Figure 1 C) in the main text via truncating the 'A' and 'B' wheels. B) Implementation of the graph from Figure 1 C) in the main text within thickness-2 by nesting wheels. Wheels corresponding to $X(f,0)$ are placed on the outside and wheels corresponding to $Z(h^T, g^T)$ are placed on the inside. The $X(f,0)$ system can be connected to a surface code in the 'A' plane. Here we only show one layer per system, but this construction can be repeated for arbitrarily many layers.
